# Assessment of genetic diversity and yield performance in Jordanian barley (*Hordeum vulgare* L.) landraces grown under Rainfed conditions

**DOI:** 10.1186/s12870-017-1140-1

**Published:** 2017-11-02

**Authors:** A. M. Al-Abdallat, A. Karadsheh, N. I. Hadadd, M. W. Akash, S. Ceccarelli, M. Baum, M. Hasan, A. Jighly, J. M. Abu Elenein

**Affiliations:** 1 Department of Horticulture and Crop Science, Faculty of Agriculture, The University of Jordan, Amman, 11942 Jordan; 2 International Center for Agricultural Research in the Dry Areas (ICARDA), P.O. Box 950764, Amman, 11195 Jordan; 3 Al-Mushaqer Regional Center, NCARE, Madaba, Jordan; 4 Consultant, Rete Semi Rurali, Via di Casignano 25, 50018 Scandicci, FI Italy; 5 Department of Plant Production and Protection, Faculty of Agricultural Technology, Al-Balqa’ Applied University, Al-Salt, 19117 Jordan; 6 Agriculture Victoria, Bioscience Research, AgriBio, Centre for AgriBiosciences, Bundoora, VIC 3083 Australia; 7 School of Applied Systems Biology, La Trobe University, Vic, Bundoora, 3083 Australia

**Keywords:** GWAS, QTL, Drought, Grain yield, Single nucleotide polymorphism

## Abstract

**Background:**

Barley (*Hordeum vulgare* L.) is a major cereal crop, which is cultivated under variable environmental conditions and abiotic stresses in marginal areas around the globe. In this study, we evaluated 150 Jordanian landraces obtained from ICARDA Gene Bank and four local checks for yield and yield components related-traits in two locations across Jordan for three growing seasons under rainfed conditions. The study aims to identify superior Jordanian barley genotypes under dry conditions, to understand the genotype × environment (G × E) interactions, to analyze stability parameters and to identify markers associated with yield and yield components under rainfed conditions.

**Results:**

The barley accessions exhibited significant variation for all traits studied. Three accessions with high yield, cultivar superiority and stability under specific environments were identified with accession G69 is the highest yielding and superior for Madaba and overall environments and G144 is the highest yielding at Ramtha. Accession G123 was high yielding in all environments and was stable across different environments. At the genetic level, the Jordanian landraces were found to be diverse with a clustering that was based on row-type. The GWAS analysis identified 77 significant markers-traits associations for multiple traits including grain yield (GY) with three significant QTLs located at 1H, 2H and 7H, which seem important for dry environments.

**Conclusion:**

Utilizing Jordanian barley landraces can effectively improve and adapt the current barley cultivars for cultivation under environmental stresses in dry regions. Utilization of markers associated with important agronomical traits and their incorporation in breeding using marker assisted selection can improve barley tolerance to drought stress.

**Electronic supplementary material:**

The online version of this article (10.1186/s12870-017-1140-1) contains supplementary material, which is available to authorized users.

## Background

The domestication of barley (*Hordeum vulgare* L.) took place prior to 10,000 B.C in the Near East region known as the “Fertile Crescent”, which extends from the southern parts of Jordan to southeastern Turkey to western Iran [1]. The first results of the domestication processes are populations known as “landraces”. Farmers had a crucial role in this crop evolution process and in the maintenance of genetic variation of landraces in their farming environment [2]. Landraces usually have limited geographic range, are diverse within particular types, are adapted to local conditions and are able to tolerate drought, diseases and pests [3]. In the case of barley, landraces are common in marginal, low-input and drought-prone environments in the Fertile Crescent and in other types of stressful environments [1, 4]. In addition to their adaption to harsh environmental conditions, they are popular among farmers for their high feed quality of both grain and straw [5]. In Jordan, barley is cultivated in areas that receive an annual rainfall between 150 and 300 mm. These areas face many challenges that are increasing because of climate change, which already resulted in an increased frequency of dry years. Barley landraces domesticated and evolved in the dry areas of the Fertile Crescent are expected to be adapted to growth-limiting factors as this adaptation contributes to their yield stability.

Molecular markers technology offers the possibility to increasing the efficiency and precision of selection in plant breeding program, especially in QTL identification, which determines the location of genes on chromosomes. Therefore, the identification of markers closely linked to important agronomic traits will allow their utilization in marker-assisted selection and thus increase the efficiency of selection in breeding programs [6, 7]. A genome wide association study (GWAS) is a QTL mapping approach that makes use of natural populations, landraces, collection of cultivars released over years and genotypes with little or no pedigree information [8]. It is used to detect marker-trait relationships based on linkage disequilibrium (LD), considering that associated marker can be utilized in future marker assisted selection. Such approach was used for the identification of novel QTLs for selected traits with practical implications in breeding programs [9–11]. For instance, two SSR markers *Ebmac415* (chromosome 2H) and *GMS21* (chromosome 1H) were associated with increased GY by 19% and 13%, respectively, in most favorable environments for *H. spontaneum* [12]. Mickelson et al. [13] detected the regions around markers *acat466* and *acag135* on chromosome 3H, *acag273* on chromosome 4H, *TB2122* on chromosome 5H, and *acgc132* and *acgt517* on chromosome 6H that appear to be related with nitrogen metabolism in barley.

The emergence of new high throughput genotyping platforms enabled the implementation of GWAS in barley [14, 15]). Using GWAS, a natural variant of the barley homolog of *Antirrhinum CENTRORADIALIS* (*HvCEN*) was identified in a diverse set of barley germplasm and was found to control flowering and environmental adaptation [9]. In another study, GWAS was used to detect QTL for heading date, plant height, thousand grain weight, starch content and crude protein content in a diverse collection of 224 spring barleys of worldwide origin [10]. GWAS in a large panel of 615 barley cultivars using a mixed linear model identified significant associations for sixteen morphologic and nine agronomic traits [11]. GWAS was applied successfully on a large panel consisting of 2417 accessions using the barley iSelect 9 k SNP assay to dissect hull cover, heading date and spike morphology [15].

The main objectives of this study were to evaluate the performance of a sample of Jordanian barley landraces accessions under rainfed and drought-prone conditions in Jordan and to identify molecular markers associated with their performance under such conditions. In addition, the tested landraces were evaluated for major agronomic traits in order to identify superior and stable lines with improved adaptation to rainfed conditions. Molecular analysis was carried out to study genetic diversity and population structure and to identify genetic markers associated with high grain yield and yield components under dry conditions by using 9 k Illumina iSelect SNP assay and the GWAS approach.

## Methods

### Plant material

The material used in this study consists of 150 accessions of barley landraces collected in Jordan and held in the Gene Bank of the International Center for Agricultural Research in the Dry Areas (ICARDA) (Additional file 1). These accessions were collected from a wide range of geographic areas across the country. Four local checks were included in the study, namely Rum (a 6-row barley cultivar developed by CIMMYT and released in 1986), Baladi (a commonly cultivated 2-row barley landrace in Jordan), Yarmouk (a 2-row barley cultivar selected from ICARDA material and released in 2004) and Mutah (a 2-row barley cultivar selected from ICARDA material and released in 2004). The seeds of local checks were obtained from the National Center for Agricultural Research and Extension (NCARE) / Jordan.

### Field trials

A total of six field trials were carried out over three growing seasons (2008–2009, 2009–2010 and 2010–2011) in farmers’ fields at Madaba (with around 350 mm long term average rainfall) and Ramtha (with around 217 mm long term average rainfall). The field trial locations are described further in Additional file 2. All field trials were in accordance with the local legislations under the direct supervision of NCARE/Jordan.

The experimental design was a partially replicated (p-rep) row-column design with 20 columns and 10 rows. The 150 accessions were replicated once, while the four checks (Rum, Baladi, Yarmouk and Mutah) were replicated 13, 13, 12 and 12 times, respectively, which resulted in 200 plots for each field trial. Plot size was 9.75 m^2^ (6 rows × 6.5 m length × 0.25 m distance between rows) planted at a seeding rate of 100 kg ha^−1^. The PRDiGGer software (http://nswdpibiom.org/austatgen/software/) was used to randomize the entries and a different randomization was used for each of the six year x location combinations. The software uses a user’s specified blocking sequence to distribute replicated treatments in a balanced way across the design to provide an unbiased estimate of the error variance.

The materials were sown on the 1st week of December in each growing season and grown under rainfed conditions. At each location, experimental plots were managed following the standard agricultural practices including weed control (hand weeding and herbicides against broad-leaved weeds) and pesticide use against main pathogens. Data on precipitation during the growing seasons were obtained from meteorological stations nearby the experimental sites (Additional file 2).

### Field data collection

The traits recorded on plot basis were: grain yield (GY in g/m^2^), as average grain weight of two one m^2^ sample randomly taken from each plot, measured after threshing and cleaning; biomass yield (BY in g/m^2^), as average straw and grain weight of two “one-m^2^” sample randomly taken from each plot; straw yield (StY in g/m^2^) obtained by subtracting GY from BY; harvest index (HI as %) calculated by dividing GY by multiplied by 100; thousand kernel weight (TKW in g), as the weight of 200 kernels taken from the bulk seed of the plot and multiplied by 5; spike weight (SW in g), as the weight of five randomly selected spikes in the plot; kernels per spike (K/S): as the number of kernels/spike counted on five randomly selected spikes in the plot; spike length (SL in cm), as the length measured from the base to the top of the spike excluding the awns. The following traits were recorded as an average of three plants randomly selected within each plot: plant height (PH in cm): measured at maturity from the ground level to the top of the spike excluding awns; awn length (AL in cm), as the length from the top of the spike to the longest awn; peduncle extrusion (PEX in cm), as the length of the section of the last internode from the ligule of the flag leaf to the base of spike. Negative values of PEX indicate that the spike, is at least partially, inside the boot; peduncle length (PL in cm), as the length from last node to the base of spike.

### Statistical analysis

Analysis of data from the un-replicated trials to estimate genotypic performance of the entries was carried out as described in Singh et al. [16], and adapted for the p-rep design in rows and columns. The spatial analysis procedure screens the best out of the nine spatial patterns described in terms of local fertility trends and spatially correlated plot errors and formed as all possible combinations of three fertility trends: fixed linear trend along rows (i.e. in column number), random cubic spline in column number, and no trend, and three plot-error structures: independent plot-errors, first order auto-regressive errors along rows, and first order auto-regressive error along rows and along columns). The selection of the most fitted model was done using Akaike information criteria and procedure described in Rollins et al. [17].

The best linear unbiased estimates (BLUE’s) of the genotype means were obtained for each environment and the trait as the spatial pattern are unique for a field in a given year, and used for assessing the genotypes performance specific to locations and stability over years. For each trial, the field heterogeneity was assessed by coefficient of variation as experimental error standard deviation divided by trial mean × 100, and statistical significance of genotypes effects was assessed by a chi-square based test, called Wald-test, when the genotypes effects were assumed fixed. For each individual trail, heritability in broad sense for each field trial was computed as genotypic variance component estimate divided by sum of genotypic variance component estimate and experimental error variance estimate, in which case the genotypes effects were assumed random as is the usual procedure.

BLUEs were used to examine the distribution of genotypes performance at individual trials using boxplots and using means and stability across location and overall the environments. The static stability of a genotype was obtained as coefficient of variation (CV) of its means (BLUEs) across the environments (CV% = standard deviation of the genotype means across environments divided by overall mean of the genotype × 100 [18]. An index, P, to measure superiority of a genotype was introduced by Lin and Binns [19], which integrates the genetic effect and G × E interaction in comparison with an ideal genotype achieving the maximum yield in each environment. Since P is at variance scale and to make comparable with the CV measure of stability, we have obtained a standardized coefficient of cultivar superiority (SCCS) for a genotype, which was computed as SCCS = square-root of P divided by mean of the genotype × 100. All the above statistical analyses were carried out using GenStat Edition 15 [20]. The codes for various analyses were organized as separate program files.

For environmental grouping based on location and overall, Restricted Maximum Likelihood Method was used to estimate variances of the genotype × site × year model. For this purpose, analysis of data was carried out based on augmented incomplete block design where contrasts of replicated checks and new un-replicated entries were estimated simultaneously assuming random genotypic values. Thereafter, heritability for each environmental grouping was computed as genotypic variance component estimate divided by sum of genotypic variance component estimate, genotype × year interaction, genotype × site interaction, genotype × year × site interaction and experimental error variance estimate, in which case the genotypes effects were assumed random as is the usual procedure.

The GGE biplot software [21] was used to exhibit specific adaptation and stability of the genotypes. Location-year combinations were considered one distinct environment as suggested by Costa and Bollero [22] and Okuyama et al. [23]. The GGE-biplot analysis of tested accessions with the distinct environments was carried out using the GGE biplot software (http://www.ggebiplot.com) for GY.

### Genotyping and molecular analysis

For genomic DNA (gDNA) extraction, barley plants were grown in a growth chamber under controlled conditions with temperature range between 22 and 24 °C and short-day photoperiod (12 h light). Total gDNA was extracted from leaf tissue collected from three-week-old plant of each accession using the CTAB (cetyltrimethylammonium bromide) method as described in Winnepenninckx et al. [24].

For genotyping, a set of 7864 independent, high confidence and gene-based SNPs markers, which are incorporated into the Illumina Infinium iSelect 9 k SNP barley array were used to genotype the accessions as described previously [9]. The genotyping assay was performed by the TraitsGenetics GmbH. (Gatersleben, Germany). Markers with allele frequency < 5% or missing data >10% were removed from further analyses.

### Cluster analysis, population structure and linkage disequilibrium

For genetic diversity assessment, SNP marker data was used to generate an un-rooted tree by using a neighbor-joining (NJ) algorithm implemented in DARwin software [25]. The genetic structure (Q) of the 154 barley lines was analyzed using 120 SNP markers distributed across the barley genome (a minimum distance of 10 cM between markers on the same chromosome). A clustering method based on a Bayesian model [26] and implemented in the STRUCTURE version 2.3.3 algorithm (available from http://web.stanford.edu/group/pritchardlab/structure.html) was employed to determine population structure. Both the length of burn-in period and the number of iterations were set at 100,000 iterations with k value in the range of 1–12. To reach the appropriate k value, the estimated delta k value was calculated for each estimated k according to Evanno et al. [27].

The LD between markers was estimated using a squared allele frequency correlation (R^2^) as described by Hedrick [28] and Weir [29] and only SNP markers with known chromosomal position were used in the estimation of LD. Linkage disequilibrium statistics were calculated per chromosome and across all chromosomes (Inter- and Intra-chromosomal LD), while the decline of LD with genetic map distance was evaluated by plotting R^2^ against the distance (cM) between markers and fitting a decay curve using the square root transformation of the equation described by Andreescu et al. [30]. The critical value at 95% of R^2^ values between pairs of unlinked loci was 0.197. The 2nd degree-loess (local regression) smoothing was calculated and plotted using SigmaPlot V.11 software (http://www.sigmaplot.co.uk/products/sigmaplot/produpdates/prod-updates1.php).

### Association analysis

Association between SNP markers and yield and yield components traits from selected individual environments, two locations and overall environments with heritability values ≥5 was analyzed using a mixed-model approach (MLM) to control type I errors, accounting for genetic relatedness or kinship (K). The K matrix is a measure of relative kinship and quantifies the probability that two homologous genes are identical by descent [31] and it was generated within TASSEL 5 ([32]; http://www.maizegenetics.net/tassel). The analysis was performed with and without taking into account the first three principle components as a covariate (Q matrix) [33, 34]) in TASSEL 5 and only markers that confirmed with both MLM and MLM-Q models were considered as true associations. In order to consider the dependency of markers due to LD, we estimated the significant cutoff for *P*-value using the method described in Li et al. [35]. The method evaluate the effective number of independent tests from large number of dependent markers based on their LD values to determine the accurate significance threshold. However, associations with *P* < 0.001 between SNP markers and traits were also considered as suggestive QTL. The map positions of the associated markers were based on previously described map reported in Comadran et al. [9]. Physical positions were optioned from “Morex_2016” map available at https://triticeaetoolbox.org/barley.

## Results

### Performance of the Jordanian barley landraces under rainfed environments

The rainfall data during the three growing seasons were collected from the nearest meteorological stations. The rainfall amounts received in the six field trials were below the long-term average of the respective locations (Additional file 2). The recorded weather data indicates the presence of variations in rainfall patterns across different locations and years indicating the prevalence of drought conditions at tested sites.

**Fig. 1:**
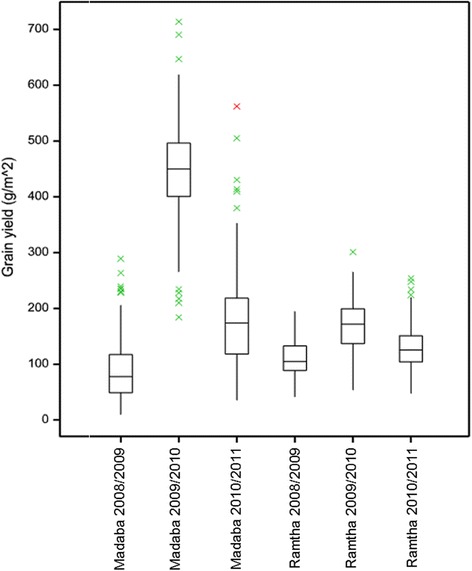
Boxplots of BLUEs of 154 Jordanian barley genotype means for GY for each of the eight trials

Combined ANOVA showed highly significant differences for location × year × genotype interactions with 4.05% of the total sum of squares attributed to genotypic effects (Additional file 3). The best spatial model screened for GY (Additional file 4) shows that out of six trials, linear trends were present in field along the rows of the layout of the trial at Madaba (2010/2011) and Ramtha (2009/2010) while auto-correlation was found in the plot-errors along rows alone at Madaba, 2008/2009 and along both directions in case of Ramtha 2010/2011. No spatial patterns could be gauzed through the nine models used, although the experimental fields showed a high degree of heterogeneity, where the field coefficient of variation varied in the range 18–52%. The Walt test assuming genotype effects fixed showed significant genotypic differences in all the trials (*P* < 0.05) except for Ramtha 2008/2009 (*P* = 0.10). However, when genotype effects were assumed random, the broad sense heritability estimates varied from 5 to 45% in four trials while zero in the remaining trials (Additional file 4). In the latter trials the estimates of variance components were zero using restricted maximum likelihood (REML) method. This may be due to lack of desired replications for the test and the check entries. Distributions of BLUEs of genotype means for GY for each trial are summarized as boxplots in Fig. 1. Of all the 6 trials, the highest means and spread of the BLUEs were observed for 2009/2010 at Madaba while the lower mean levels were observed for Madaba in 2008/2009.

On the another hand, environmental grouping based on location and overall was considered and the Restricted Maximum Likelihood Method was used to calculate their heritability values, which was 20.26% for overall and varied from 8.2 to 12.4% in Madaba and Ramtha, respectively (Additional file 3).

The BLUEs were averaged over the years at each locations and also overall the trials and are presented in Table 1 (full list of genotypes and their statistics are provided in Additional file 5) for top fifteen genotypes along with their measures of stability, CV% and genotype superiority (SCCS). Denoting the genotypes/accessions 1–154 by G1 – G154, a spectacular accession was G69, which was the highest yielding overall (259 g/m^2^) and at Madaba (389 g/m^2^). Based on the cultivar superiority index (SCCS), G69 is also ranking first in overall and Madaba (Table 1). The highest yielding genotype at Ramtha was G144 with GY mean value of 195 g/m^2^. Accession G123 was identified within the top 15 genotypes at Madaba, Ramtha and overall and it was stable at Madaba and overall when compared with Ramtha where it was considerably less stable (Table 1). There is a very high, though negative, correlation between mean and the superiority index SCCS (Additional file 5), which makes SCCS redundant for any additional advantage of the genotype beyond mean over the environments. Correlation between mean and CV%, as a stability measure, was found much lower, ranging from −0.35 to 0.07, and significant at Madaba. This provides a scope for identifying genotypes which are high yielding (or superior) as well as stable.

**Table 1:** Means, coefficient of variation (CV %) and standardized cultivar superiority over years of the best linear unbiased estimates (BLUEs) of GY (g/m^2^) of the top 15 genotypes

	Madaba				Ramtha				Overall environments			
Rank	Accession	Mean	CV%	SCCS%	Accession	Mean	CV%	SCCS%	Accession	Mean	CV%	SCCS%
1	**G69**	388.7	73.12 (58)^*^	32.9 (1)	G144	194.8	34.63 (96)	22.01 (1)	**G69**	259	89.01 (118)	42.06 (1)
2	**G60**	351.5	43.29 (9)	38.59 (2)	G99	187.2	33.14 (89)	30.55 (5)	G106	249	68.03 (57)	42.23 (2)
3	**G25**	350.7	24.62 (1)	42.23 (6)	**G32**	183.7	12.35 (13)	28.56 (3)	**G30**	247	55.53 (25)	47.88 (6)
4	**G30**	341.4	41.6 (7)	44.46 (7)	G128	181.8	22.44 (44)	30.62 (6)	**G87**	246	68.35 (58)	42.37 (3)
5	G100	337.2	81.59 (77)	47.46 (11)	G48	181.7	22.57 (45)	26.97 (2)	**G60**	246	61.05 (35)	45.61 (4)
6	**G87**	335.8	61.65 (33)	39.29 (3)	G89	179.7	16.64 (24)	29.05 (4)	**G123**	246	48.96 (10)	47.76 (5)
7	**G106**	332.9	64.72 (39)	40.59 (4)	G8	177.4	60.87 (149)	38.24 (11)	**G32**	241	52.82 (18)	52.86 (7)
8	G41	329.9	68.01 (48)	41.72 (5)	**G34**	172.4	54.34 (145)	38.29 (12)	G85	235	87.55 (115)	55.46 (12)
9	**G85**	324.7	86.64 (86)	50.67 (13)	G139	171.9	2.19 (1)	36.32 (9)	**G34**	232	69.9 (63)	54.23 (9)
10	**G123**	323.8	37.84 (5)	46.31 (9)	G59	171	48.06 (132)	36.08 (8)	**G25**	228	64.48 (44)	55.97 (14)
11	G2	320.7	73.07 (57)	44.62 (8)	G73	169.9	41.33 (119)	34.36 (7)	**G132**	227	76.57 (85)	58.56 (16)
12	**G18**	320.1	104.73 (129)	52.98 (17)	**G123**	168.4	33.52 (91)	42.61 (23)	G93	226	63.53 (40)	53.8 (8)
13	G88	320	41.14 (6)	50.89 (14)	G39	166.3	29.66 (71)	40.66 (19)	**G18**	225	105.59 (145)	60.75 (21)
14	**G132**	319	67.88 (47)	51.84 (15)	G95	165.9	13.88 (15)	42.15 (21)	G36	223	76.94 (87)	54.86 (11)
15	G22	314.1	62.45 (36)	47.11 (10)	G131	165.2	34.89 (97)	38.74 (14)	G66	222	65.79 (47)	60.01 (18)

Accessions in bold are present across at least in two different environments

^*^Number between bracts are representing stability ranks

**Fig. 2:**
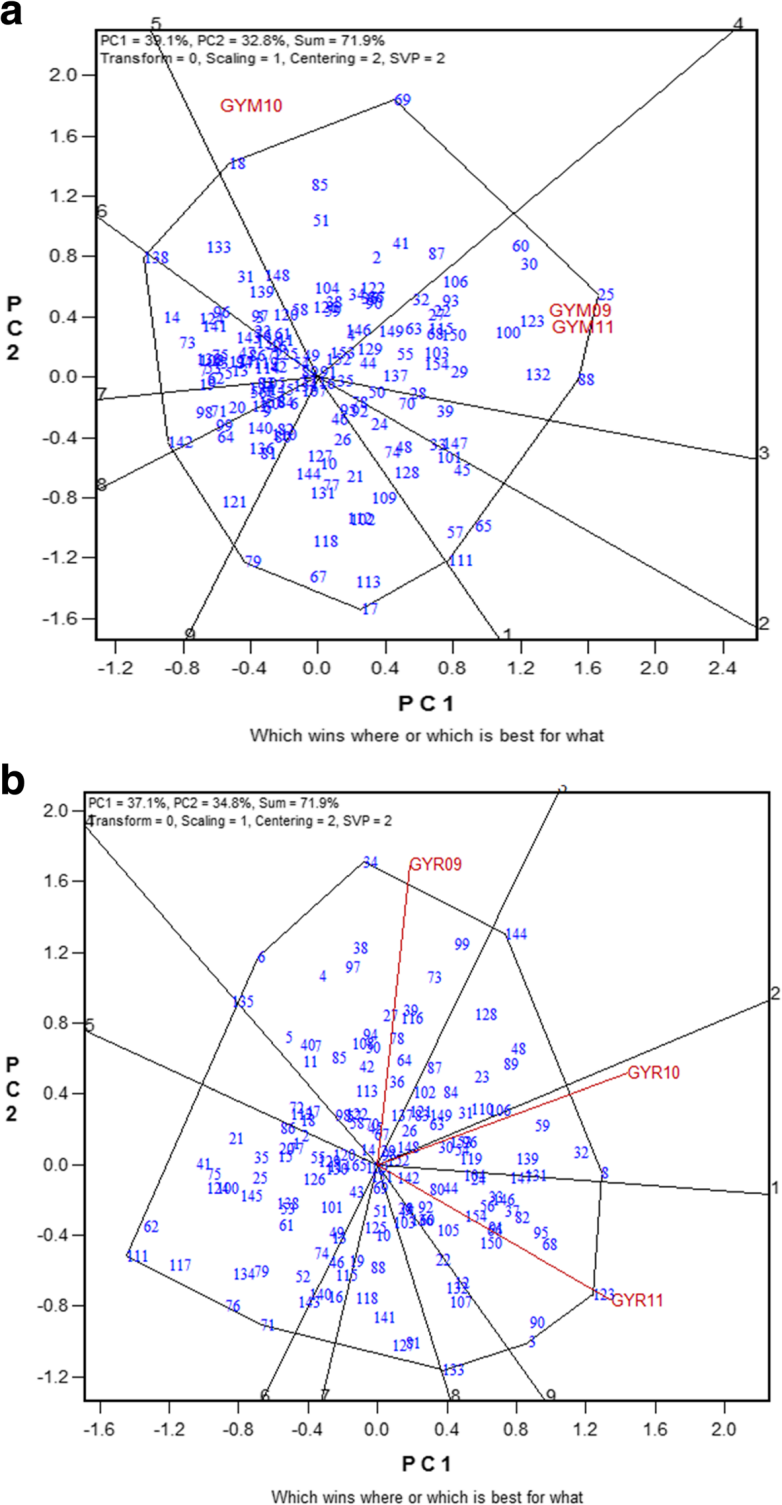
GGE biplot of grain yield (GY) GGE biplot analysis for 154 Jordanian barley genotypes used in this study grown in **a** Madaba (M) and **b** Ramtha (R) in 2008/2009 (09), 2009/2010 (10) and 2010/2011 (11) growing seasons

The broad and specific adaptation of accessions to tested environments for GY was studied by using the GGE biplot analysis based on location-year combinations (Fig. 2). Using the means in each location for available years and considering all the genotypes, the first two principal components (PC) accounted for a total of 71.9% variation in both locations. In Madaba, the GGE biplot analysis showed that the growing seasons were divided into two sections where the 2008/2009 and 2010/2011 growing seasons fall in the same section and the 2009/2010 season was in another section and the tested accessions were scattered into nine sections (Fig. 2a). G25 had the highest GY in Madaba in 2008/2009 and in Madaba in 2010/2011, while G69 had the highest GY in Madaba in 2009/2010 (Fig. 1a). In Ramtha, the GGE biplot showed that the growing seasons were divided into three sections and the accessions into nine sections. G34 had the highest GY in Ramtha in 2008/2009, while G8 had the highest GY in Ramtha in 2009/2010 and G123 (Fig. 2b). The biplot analysis results were in general agreement with the BLUEs values in terms of entries performance across environments.

### Association between the agronomic traits

Grain yield showed significant (*P* ≤ 0.01) correlation with BY and StY in both locations (Madaba and Ramtha) and overall environment (Table 2). TKW was significantly correlated at Ramtha but not at Madaba. The other traits that showed significant correlations with GY were PH at both locations and overall; with PEX and AL at Madaba and under overall (Table 2). Correlation heat maps are given for the individual trials for all possible variables where data were recorded (Additional file 6). In general, GY showed a strong positive correlation with BY and StY across different trials.

**Table 2:** Correlation coefficients between GY and other traits based on154 barley genotypes using means over the available years at two locations and overall

Trait	Madaba	Ramtha	Overall
BY	0.86^*^	0.82	0.84
StY	0.66	0.57	0.69
SW	−0.42	0.04	−0.28
TKW	0.04	−0.32	0.01
HI	0.73	−0.04	0.40
K/S	0.29	−0.10	0.06
SL	0.36	0.28	0.34
PH	0.84	0.34	0.71
PL	0.77	0.26	0.58
PEX	0.68	0.08	0.55
AL	0.28	0.05	0.17

^*^Critical values of correlation are based on 154 observations = 0.158 at 5%, 0.207 at 1% and 0.263 at 0.1% levels of significance

### Marker-trait associations

**Fig. 3:**
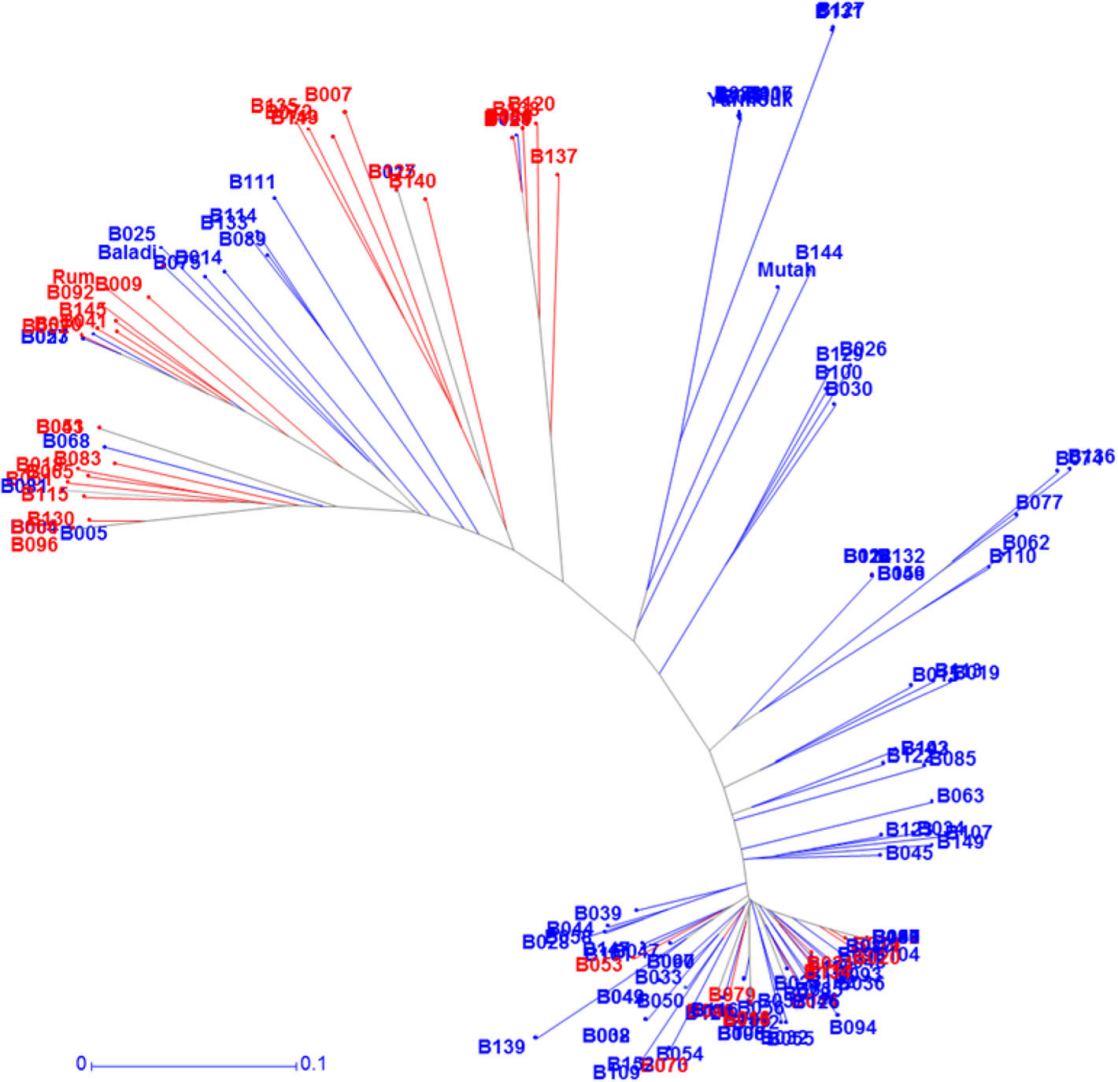
Unrooted neighbour joining tree of 154 Jordanian barley accessions used in this study using UPGMA clustering method. (red for 2-row and blue circle for 2-row)

For the GWAS analysis, filtering the 9 K SNP markers data resulted in 4956 polymorphic SNPs. Chromosome 5H had the largest number of markers (629), while chromosome 4H carried the smallest number with 253 SNPs. Those SNPs spanned a total genetic distance of 989.9 cM with an average of one marker every 0.3 cM (data not shown). The population structure resulted in two distinct sub-populations (Additional file 7). The first sub-population involved 86 genotypes with *Fst* value equal to 0.67 while the second one has the rest (68 genotypes) including the four checks with *Fst* value of 0.29. Using DARwin software, an unrooted NJ tree was built describing the relatedness between Jordanian accessions (Fig. 3). The clustering analysis showed that the panel was diverse and separated into several clades where a large one contained the majority of “2- row” accessions (63) and 12 “6-row” accessions that were highly related. The rest of 6-row barley accessions (29 were identified in distinct clades that included few 2-row accessions. Other subgroups consisted exclusively from 2-row accessions with two distinctive subgroups with the first one including the Baladi check and the second including Mutah and Yarmouk cultivars (Fig. 3).

For LD decay analysis, the LD decayed sharply to an R^2^ value of about 0.2 in less than 2 cM (Additional file 8). The effective number of independent markers following Li et al. [35] was 431.2 (~14.7%). This adjustment shift the Bonferroni correction from 1.7 × 10^−5^ to 1.6 × 10^−4^. The GWAS analysis revealed eleven marker-trait associations (MTA) significantly associated with yield and yield components across the tested environments with heritability values ≥5 (Table 3) and another 77 suggestive associations at *P* < 0.001 (Additional file 9). QQ plots that compare the expected and the observed *P* values for all traits are shown in Additional file 10. Those eleven MTAs represents eight different QTL of which the marker *SCRI_RS_1971* on chromosome 5H (168.5 cM) showed association with GY in Madaba 2008/2009 (Table 3). Another interesting QTL located on chromosome 7H (1.6 cM) was associated with K/S in Madaba (Table 3) and K/S in overall and with GY Madaba 2008/2009 environments (Additional file 9). Another important QTL on chromosome 1H also showed association with BY and SL in Madaba 2008/2009 and Madaba 2010/2011, respectively (Table 3). Another QTL was also identified at 1H (133 cM) that was associated with many yield components traits in overall environments (Additional file 9). The other QTLs were found on chromosomes 2H, 4H, 5H and 7H for, “BY and HI”, TKWT, SW and “PH, StY and SL”, respectively (Table 3). Of those, QTL on 2H (~106 cM)*,* which was significantly associated with BY and HI in Madaba 2010/2011and with HI in Ramtha 2009/2010 and, was also associated with other yield components traits across different environments but at lower *P*-value (Additional file 9). The remaining suggestive MTAs were distributed on all chromosomes and confirmed many of the significant QTLs.

**Table 3:** Significant markers associated with yield and yield components identified in this study

Environment	Trait	Marker	Chr	Pos_Gen	Pos_Pys	p-value	R^2^	Allele	Add	MAF
Madaba 2010/2011	SL	*SCRI_RS_155997*	1H	104.7	528,880,379	4.97889E-05	11.211646	A:A	0.753018074	29.22077922
Madaba 2008/2009	BY	*BOPA1_7527–1618*	1H	NA	536,146,950	6.127E-05	13.5	A:A	−325.39	11.7
Ramtha 2009/2010	HI	*BOPA1_3180–1771*	2H	106.4	704,365,928	4.489E-05	11.0	A:A	5.60	33.1
Madaba 2010/2011	BY	*SCRI_RS_15050*	2H	106.8	705,595,274	1.053E-04	9.8363383	C:C	−0.274154089	42.85714286
Madaba 2010/2011	HI	*BOPA2_12_30459*	2H	106.8	NA	1.238E-04	9.8	C:C	−0.28	43.5
Madaba 2008/2009	TKWT	*SCRI_RS_152271*	4H	NA	641,600,274	3.41203E-05	10.233882	C:C	6.862072971	17.53246753
Madaba 2008/2009	GY	*SCRI_RS_1971*	5H	168.5	NA	2.364E-05	14.8	C:C	−19.60	38.3
Madaba	SW	*SCRI_RS_169596*	5H	NA	556,291,232	9.038E-05	9.8	G:G	3.87	18.8
Madaba	K/S	*SCRI_RS_229445*	7H	1.6	5,199,041	9.875E-05	9.7	G:G	21.13	43.5
Madaba 2010/2011	PH	*SCRI_RS_147695*	7H	NA	15,620,701	3.679E-05	14.3	C:C	−5.39	7.8
Madaba 2010/2011	StY	*SCRI_RS_47206*	7H	15.4	19,588,621	8.47071E-06	17.17131	G:G	−11.88941784	44.15584416

Abbreviations: Chromosome (Chr); Position (Pos); Genetic (Gen); Physical (Pys); Additive Effect (Add); Minor Allele Frequency (MAF); Madaba (M); Ramtha (R); Grain yield (GY); Biomass yield (BY); Harvest index (HI); kernels per spike (K/S); Peduncle extrusion (PEX); Plant height (PH); Peduncle length (PL); Spike length (SL); Spike weight (SW)

## Discussion

The knowledge of genetic diversity available in germplasm collections or breeding material helps the breeders to plan their programs for specific environments using targeted traits and molecular markers. Hence, this study was conducted to evaluate 150 Jordanian barley landraces and 4 improved varieties used as checks, in different dry areas of Jordan. Significant differences between accessions in yield and yield components were identified across different environments. This indicates the presence of variability in the agro-morphological traits within these accessions, which provide ample scope for selecting superior accessions by plant breeders. This is somehow expected, since Jordan is considered a part of the center of origin for wheat and barley [36, 37]. Similar results were observed by Ceccarelli et al. [38], who revealed significant genetic variation among 10 traits for 70 barley landraces, 60 of which were collected from Syria and 10 from Jordan. Therefore, the landraces analyzed in this study can be considered as a reservoir of genes that plant breeders need in their barley-breeding program.

Accessions that ranked first for a number of traits were significantly superior to the check varieties under rainfed conditions. The range for GY between accessions varied with location and the mean GY was in the range of 130–389 g/m^2^ at Madaba and 71–195 g/m^2^ at Ramtha (Additional file 5). Furthermore, some accessions performed well in some environments where they had the highest value for one trait but the lowest value for other traits (data not shown). Similar results were found by Rodriguez et al. [39], who found that variations was attributed mainly to environment effect (67.1%), genotypes effect (5.3%) and G × E interaction (26.7%). This is expected in such very dry sites, as rainfall (amounts and its distribution) and temperature differs from year to year in the same location. Similar results were obtained by Rizza et al. [40], Sudaric et al. [41] and Mohammadi and Amri [42]. Thus, to reduce the magnitude of the interaction it was suggested by Mohammadi et al. [43] to divide the testing area into sub-regions to avoid inconsistent environmental conditions prevailed across locations.

In this study, accession G69 gave the highest yield in Madaba and overall environments (Table 1). This is somehow expected knowing that G69 was collected from a semi-humid area in northern parts of Jordan where annual rainfall is higher than 350 mm (Additional file 1). Accession G139 was highly stable and performed well in Ramtha location indicating the suitability of this accession to arid regions in north of Jordan. Accession G123 showed good performance across different environments and was highly stable in Madaba and overall indicating its suitability for dry environments (Table 1). All of these good performing accessions were of 2-row barley type, which are known to be adapted to drought conditions. The “Baladi” traditional local barley landrace in Jordan and in neighboring countries with similar drought conditions have a 2-row type, which is preferred by local farmers. The GGE biplots provided further support for the adaptation of the identified superior genotypes to specific locations (Fig 2).

In this study, SNP markers were used to analyze the level of diversity among and within Jordanian barley landraces. A similar approach was used previously to study genetic diversity in barley lines from different origins [44, 10]. Genetic variation among Jordanian accessions identified to be large and several subgroups were observed based on row-type. A subgroup included the majority of accessions with 2-row barley type that show high level of relatedness, while distal subgroups included the 6-row accessions and distinct 2-row accessions like the Baladi subgroup (Fig. 3). On the other hand, the population structure analysis identified two subgroups that where considered highly structured reflecting the impact of material origin and row type. The separation of Jordanian barley landraces according to head type (two and six rows) is in general agreement with the results of Pasam et al. [10] and Cuesta-Marcos et al. [45]. The molecular analysis did not reflect the origin of the collection sites for Jordanian barley landraces (data not shown). This might be because these landraces are adapted to a wide range of agro-ecological conditions and the probability of seed exchange between farmers. Russell et al. [46] found that landraces collected from Jordan and southern Syria clustered together indicating high similarity at the genetic level. In the contrary, Varshney et al. [47] found that the genetic diversity of barley accessions used in their study was not completely related to the geographic distribution.

To further elaborate on the assumptions extracted from the phenotypic and genotypic data of the tested Jordanian accessions, GWAS was carried out in order to identify QTLs associated with yield and yield components under rainfed conditions. Selecting the best *P*-value cutoff that control for both type I and type II errors in GWAS analyses is always challenging. Using the naïve false discovery rate (FDR) methods can lead to false negative outcomes as those methods were designed for independent tests while SNPs are dependent due to genetic linkage [35]. To avoid FDR stringency, previous researches used suggestive *P*-value cutoff [48–50]; FDR cutoff at higher values up to 0.25 [51, 52]; or considered clusters of markers with R^2^ values >0.95 as dependent and can be represented by one marker [53, 54]. In this research, we calculated the number of independent test [35] followed by Bonferroni correction to accurately control for false negative results.

The GWAS analysis detected eleven significant SNP-trait associations for yield and it components (Table 3), while another 77 suggestive associations were reported in Additional file 9. These numbers are considered relatively close to other GWAS barley studies for yield and yield components under stress conditions [55, 56]. Furthermore, several QTLs identified were previously described in other related studies [47, 55, 56]. For instance, the QTL associated with BY, StY and K/S at overall detected on chromosome 1H (position: 133 cM) coincided with a previously described QTL in Mora et al. [57] and Varshney et al. [47]. In another case, Wehner et al. [56] previously detected a QTL for BY on chromosome 5H (167.7 cM) in a region close to the GY QTL in Madaba 2008/2009 while they also detected a QTL associated with BY on 5H (position: 139.1 cM) that is considerably near a GY QTL identified in Ramtha 2009/2010 (additional file 9). The QTL located at 7H (position: 1.6 cM) associated with K/S at Madaba was also reported to be associated with grain number in Ingvordsen et al. [55]. Interestingly, QTLs identified on 2H (Position: 106–108 cM), and associated with several traits across different environments was also reported in Ingvordsen et al. [55] and von Korff et al. [58]. The QTL located at 5H (position 44 cM) associated with GY in Ramtha and other yield component across all environments was reported in Wehner et al. [56]. This region includes several stress-related genes including an Abscisic acid-inducible protein kinase, which is known to mediate drought responses in cereals [59]. This could indicate the existence of conserved drought tolerance mechanisms since early domestication knowing that wild barley from Jordan is believed to be the primary ancestors in modern cultivated barley [60].

Nevertheless, QTLs identified in this study did not show clear consistency across the tested environments. This is in general agreement with the results of Varshney et al. [47] and Maccaferri et al. [61] and, who attributed this inconsistency to the existence of different genetic systems governing drought tolerance under dry field conditions. In addition, studying complex traits such as yield under dry environments using GWAS and highly structured germplasm seems to be less informative in some instances when compared with bi-parental populations [47].

## Conclusion

In conclusion, we observed a high level of variation for all of the agro-morphological traits measured in a collection of Jordanian barley landraces evaluated for three years in drought prone locations. Such variation was further confirmed at the molecular level using SNP markers analysis, which demonstrates the existence of considerable genetic variability among these accessions. Marker trait association identified highly significant and suggestive major QTLs for yield and yield components under dry environments located at 1H (position: 133 cM), 2H (position: 106 cM) and 5H (position: 44.2 cM). Finally, the superior accessions identified in this study and their respective QTLs can be used in barley breeding programs and marker assisted approach to improve productivity and stability under dry field conditions in specific locations in marginal areas of Jordan and across arid region in the world.

## Additional files

Additional file 1: ICARDA Gene bank information of 154 Jordanian barley genotypes used in this study. (XLSX 21 kb)

Additional file 2: Characteristics of field locations, actual total rainfall (mm on monthly basis), number of rainy days and long-term rainfall average in the tested sites over three growing seasons. (XLSX 10 kb)

Additional file 3: Combined analysis of variance for grain yield of barley landraces accessions and Restricted Maximum Likelihood Method estimated variances for locations and overall environments. (XLSX 14 kb)

Additional file 4; Genotypic coefficient of variation, heritability, coefficient of variation and *P*-value for grain yield of barley accessions evaluated in eight environments. (XLSX 9 kb)

Additional file 5: Means, coefficient of variations (CV%) and standardized cultivar superiority over applicable years of the best linear unbiased estimates (BLUEs) of GY (g/m2) of all tested genotypes. (XLSX 32 kb)

Additional file 6: Correlation heat maps between selected triats of all tested genotypes for each indvidual field trial. (XLSX 166 kb)

Additional file 7: Population structure of 154 Jordanian barley genotypes used in this study. (XLSX 34 kb)

Additional file 8: Decline of Genome-wide LD (as measured by R^2^) against the genetic distance (in cM). (XLSX 109 kb)

Additional file 9: Suggestive markers associated with yield and yield components identified in this study. (XLSX 20 kb)

Additional file 10: QQ plots for all tested triats in this study. (PDF 360 kb)

Additional file 11: Genotypic data of Jordanian barley landraces and four local checks used in this study. (XLSX 2582 kb)

## Abbreviations

AL: Awn length
BLUE’s: Best linear unbiased estimates
BY: Biomass yield
CV: Coefficient of variation
G × E: Genotype × Environment
GWAS: Genome wide association study
GY: Grain yield
HI: Harvest index
K: Kinship
K/S: Kernels per spike
LD: Linkage disequilibrium
MLM: Mixed-model approach
PC: Principal components
PEX: Peduncle extrusion
PH: Plant height
PL: Peduncle length
p-rep: Partially replicated
QTL: Quantitative triat loci
R2: Squared allele frequency correlation
SCCS: Standardized coefficient of cultivar superiority
SL: SPIKE length
StY: Straw yield
SW: Spike weight
TKW: Thousand kernel weight
