# Supplementary figures and images for: Assessment of genetic diversity and yield performance in Jordanian barley (Hordeum vulgare L.) landraces grown under Rainfed conditions
Source: BMC Plant Biol. 2017 Nov 2;17:191. doi: 10.1186/s12870-017-1140-1 (PMC5668982; doi:10.1186/s12870-017-1140-1)

**MLM**

AL

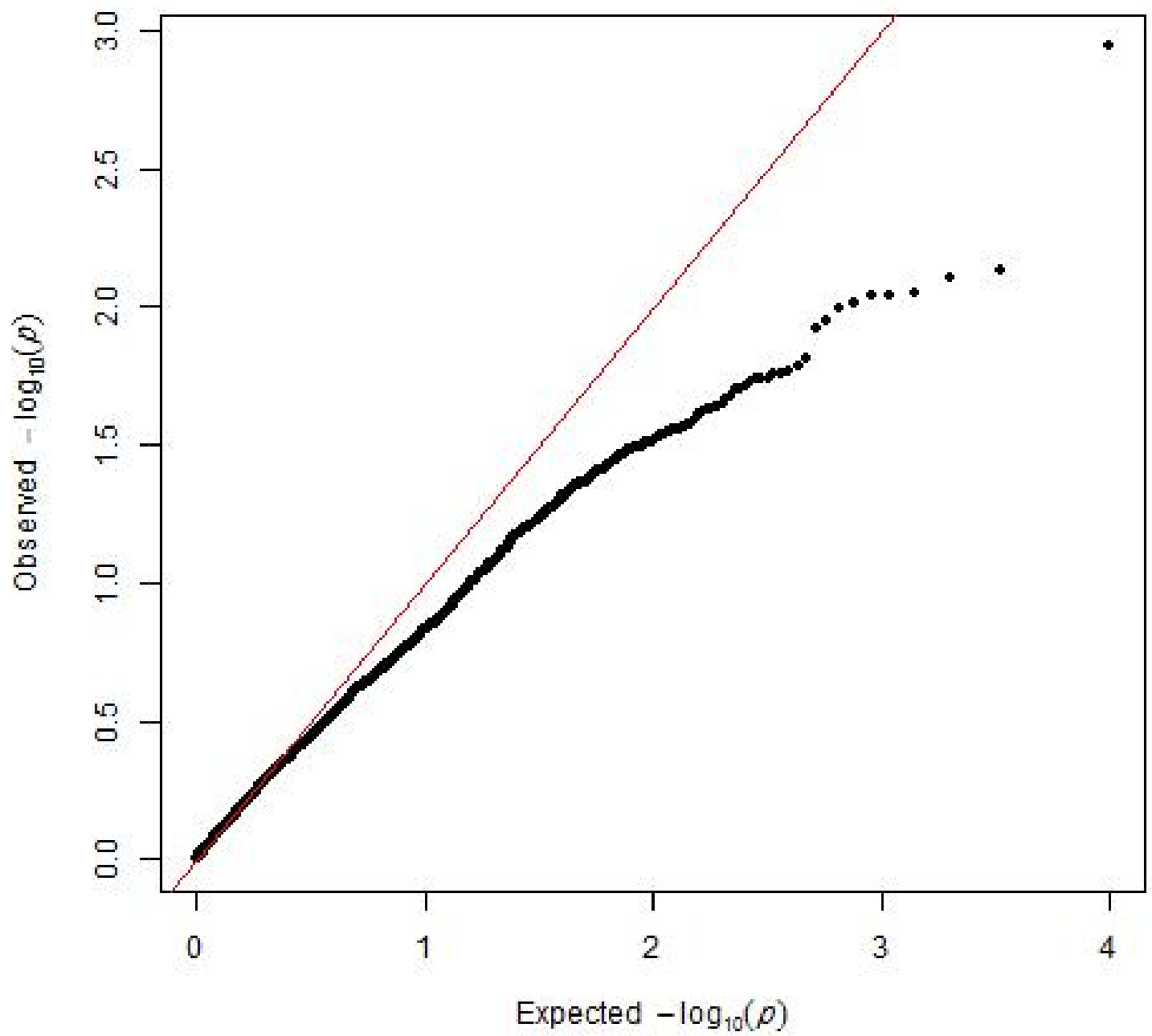

BY

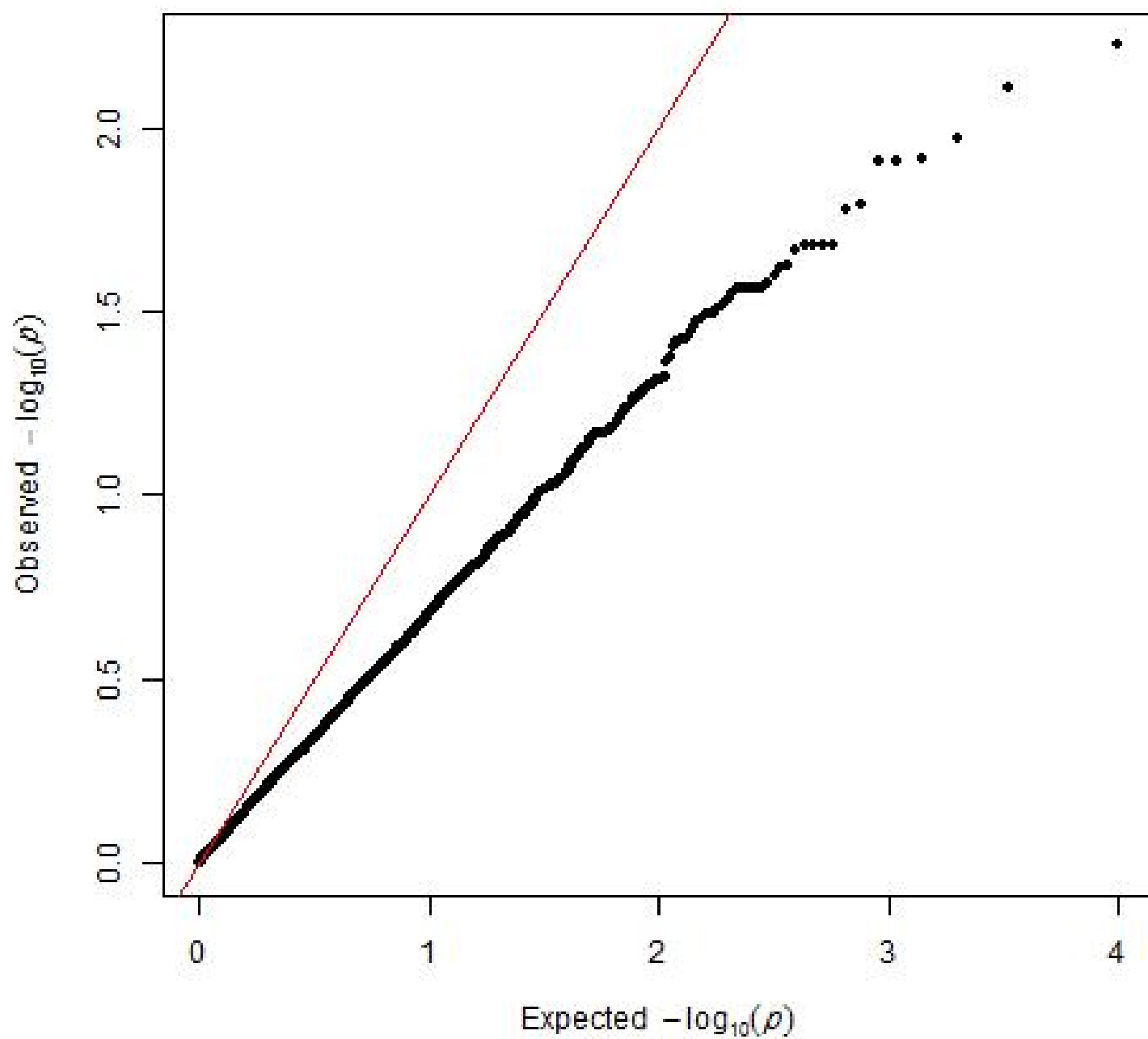

GY

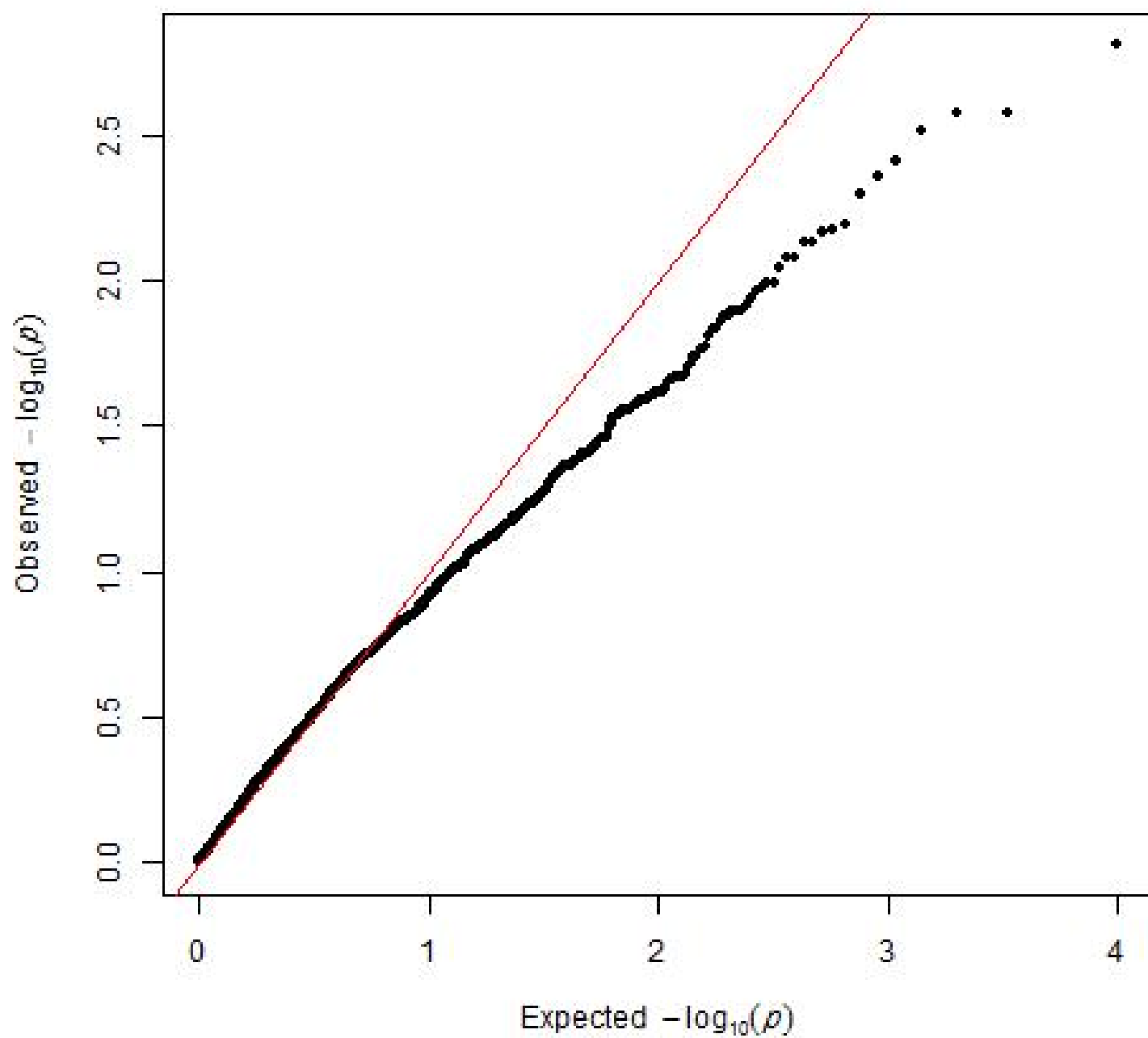

HI

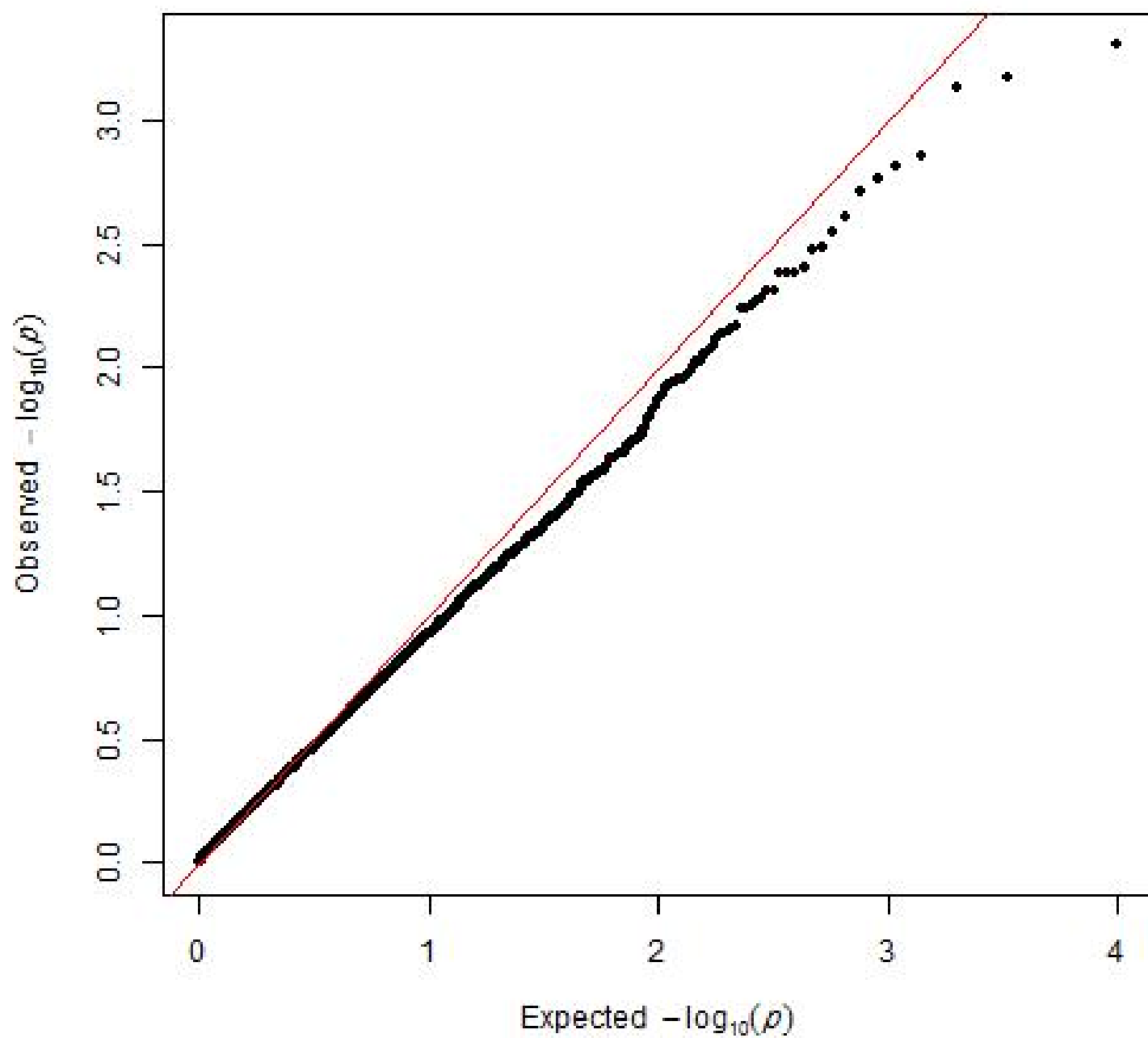

K-S

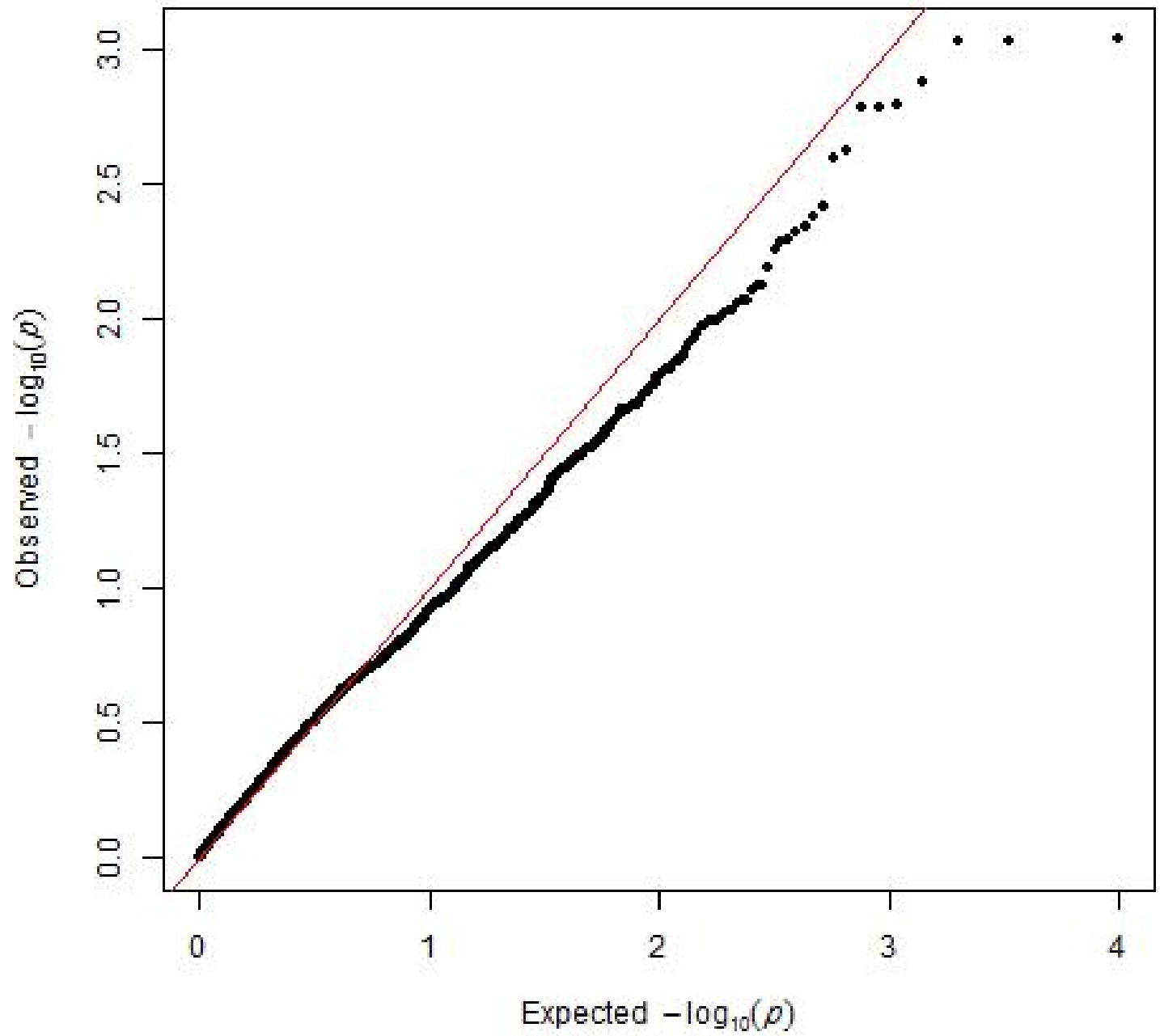

PEX

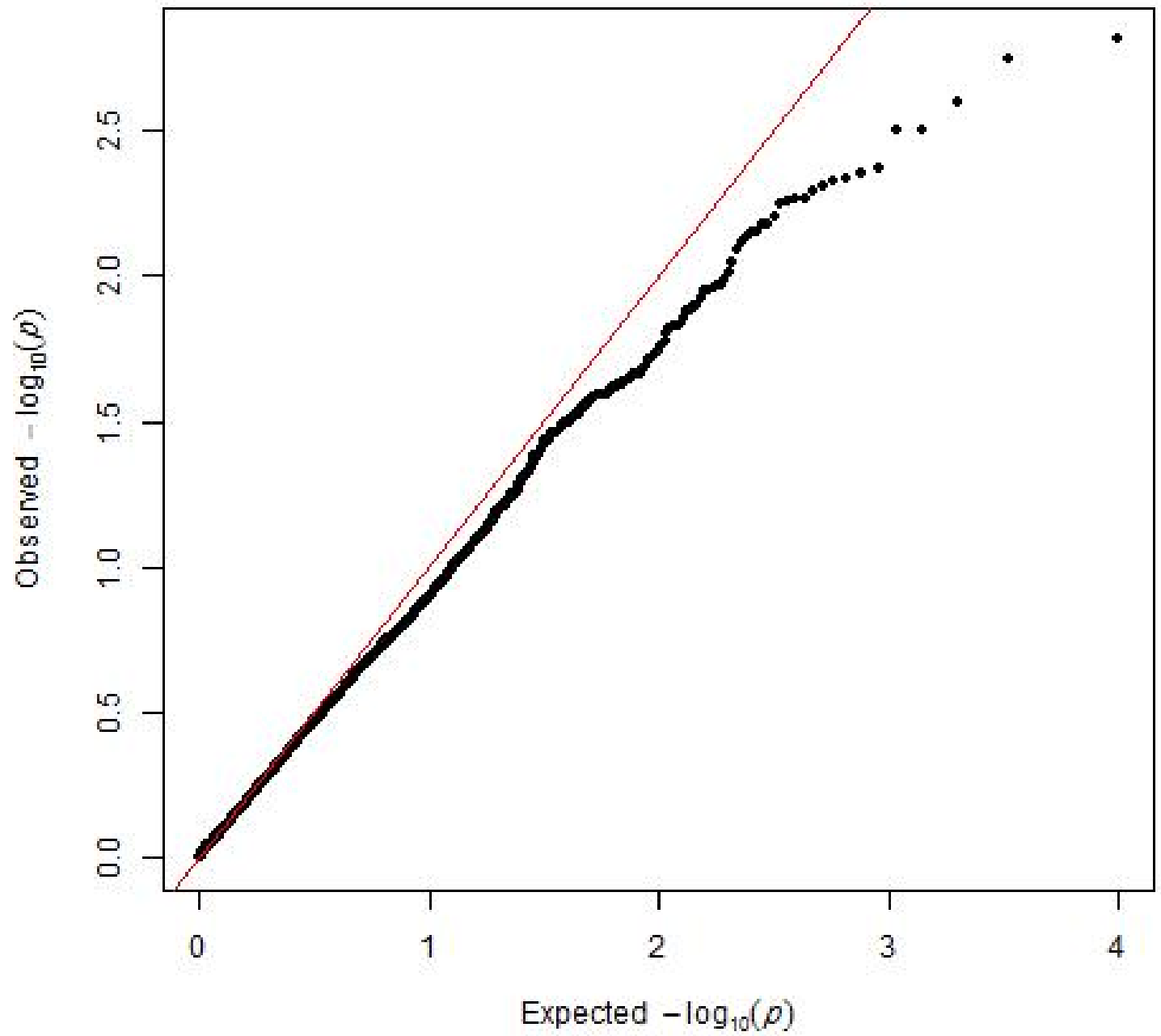

PH

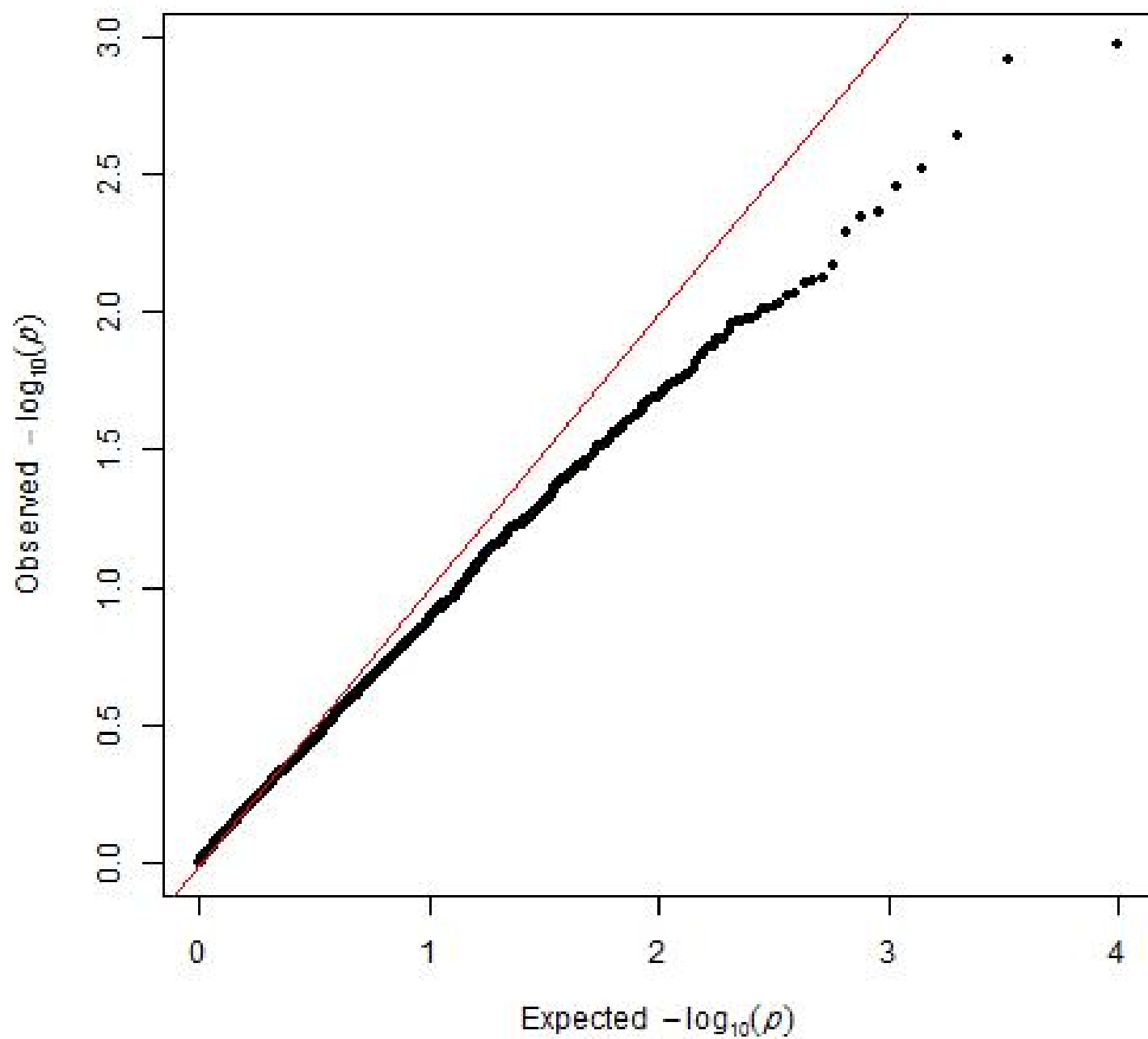

PL

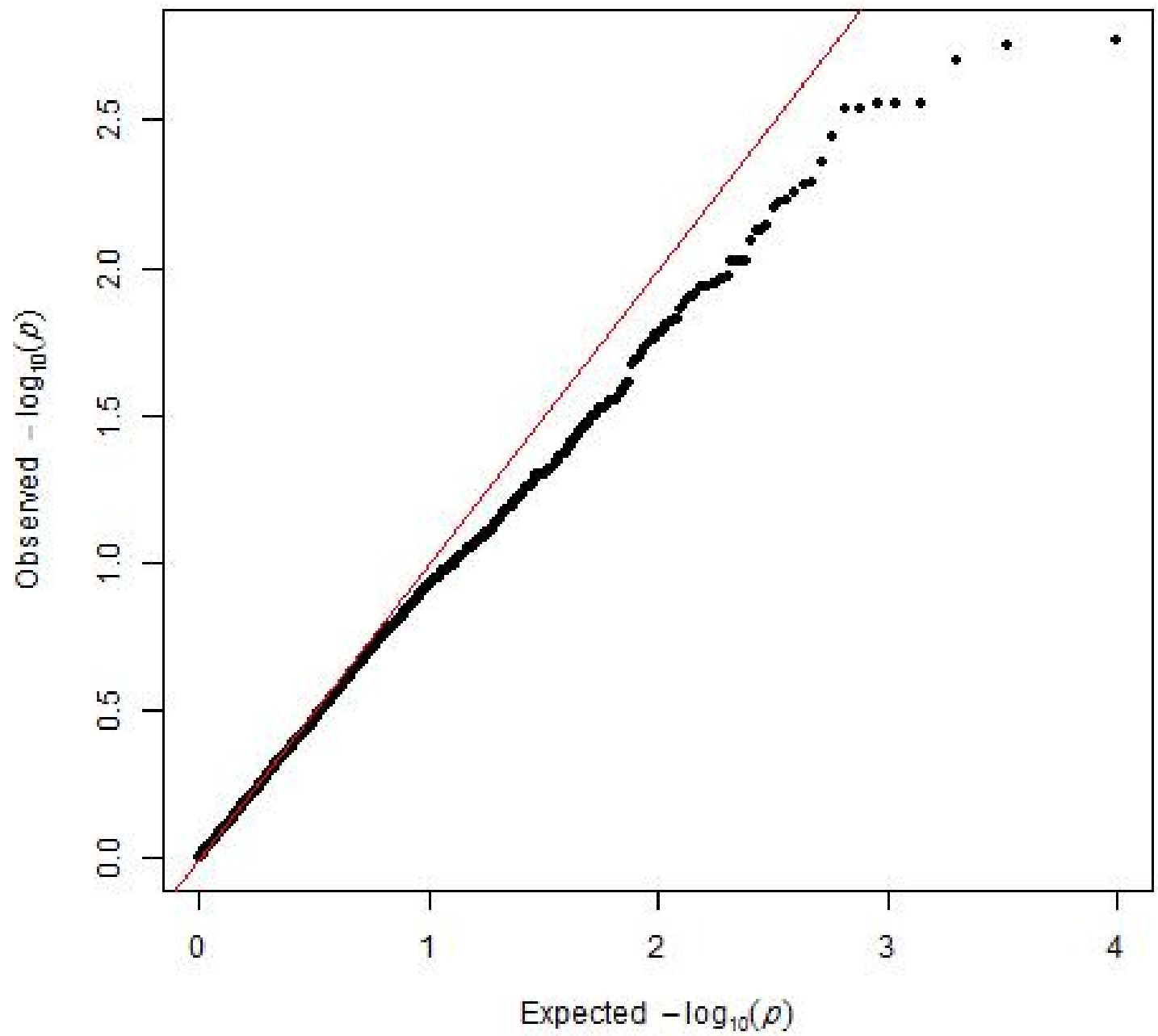

SL

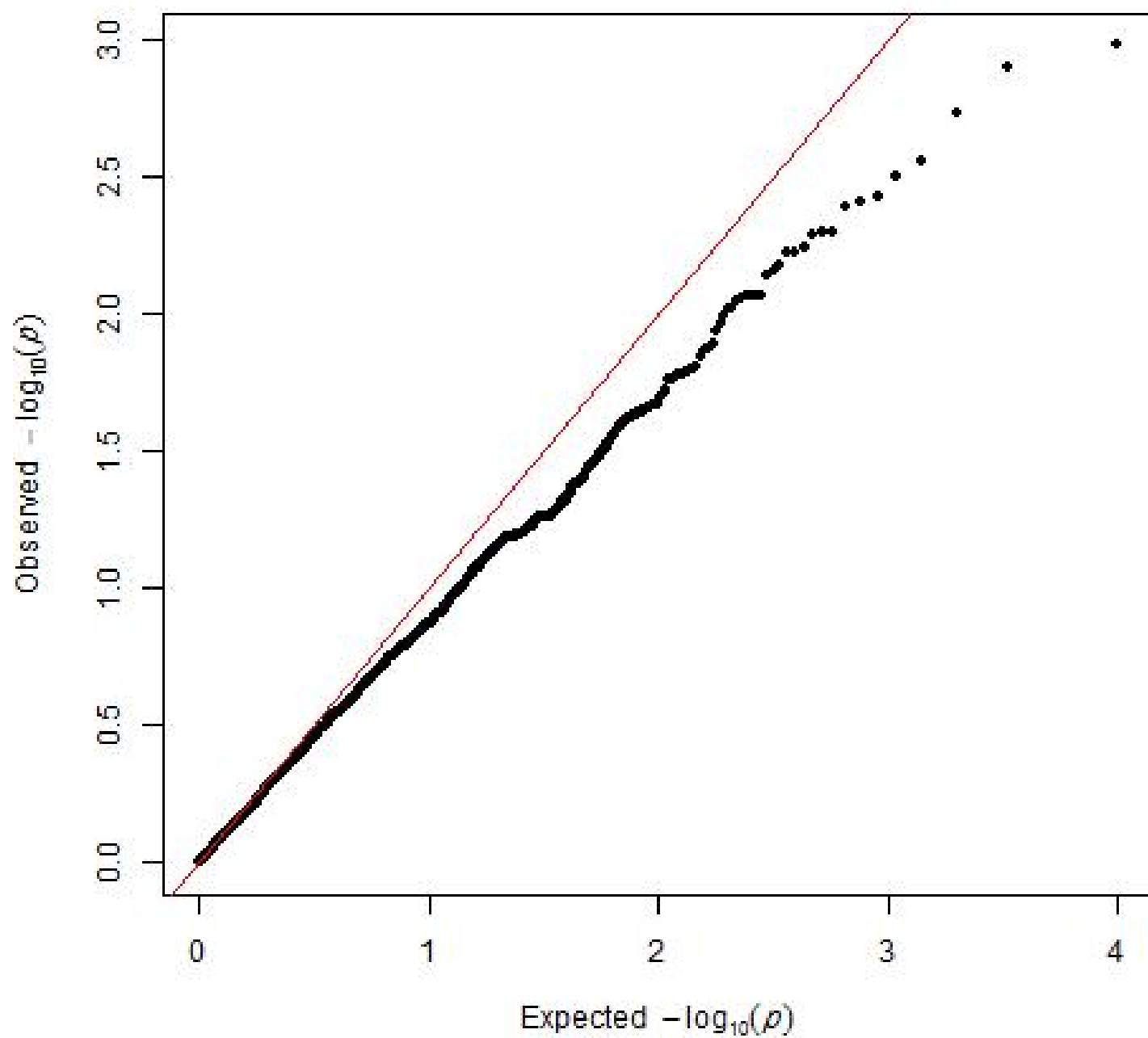

StY

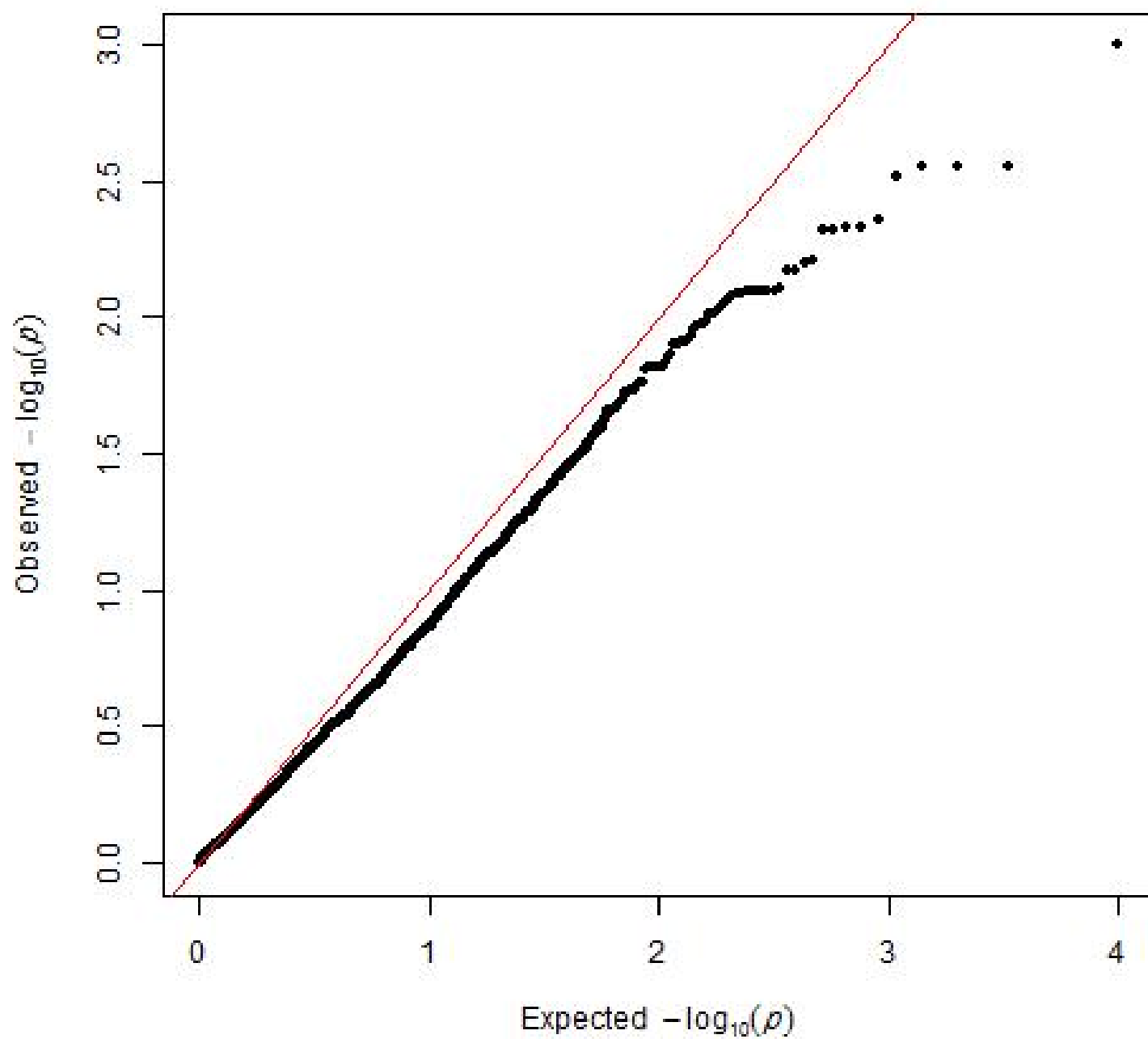

SW

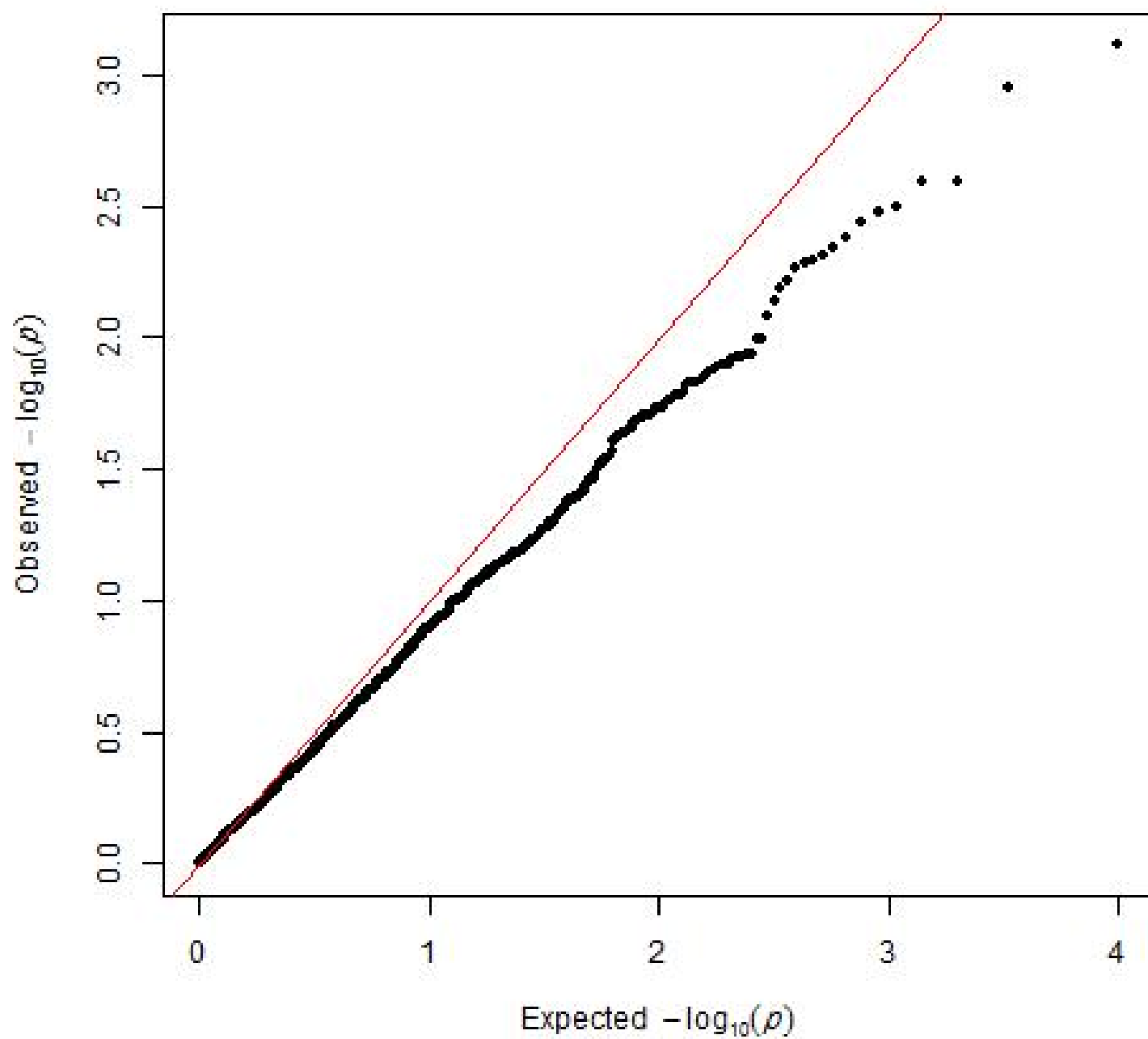

TKWT

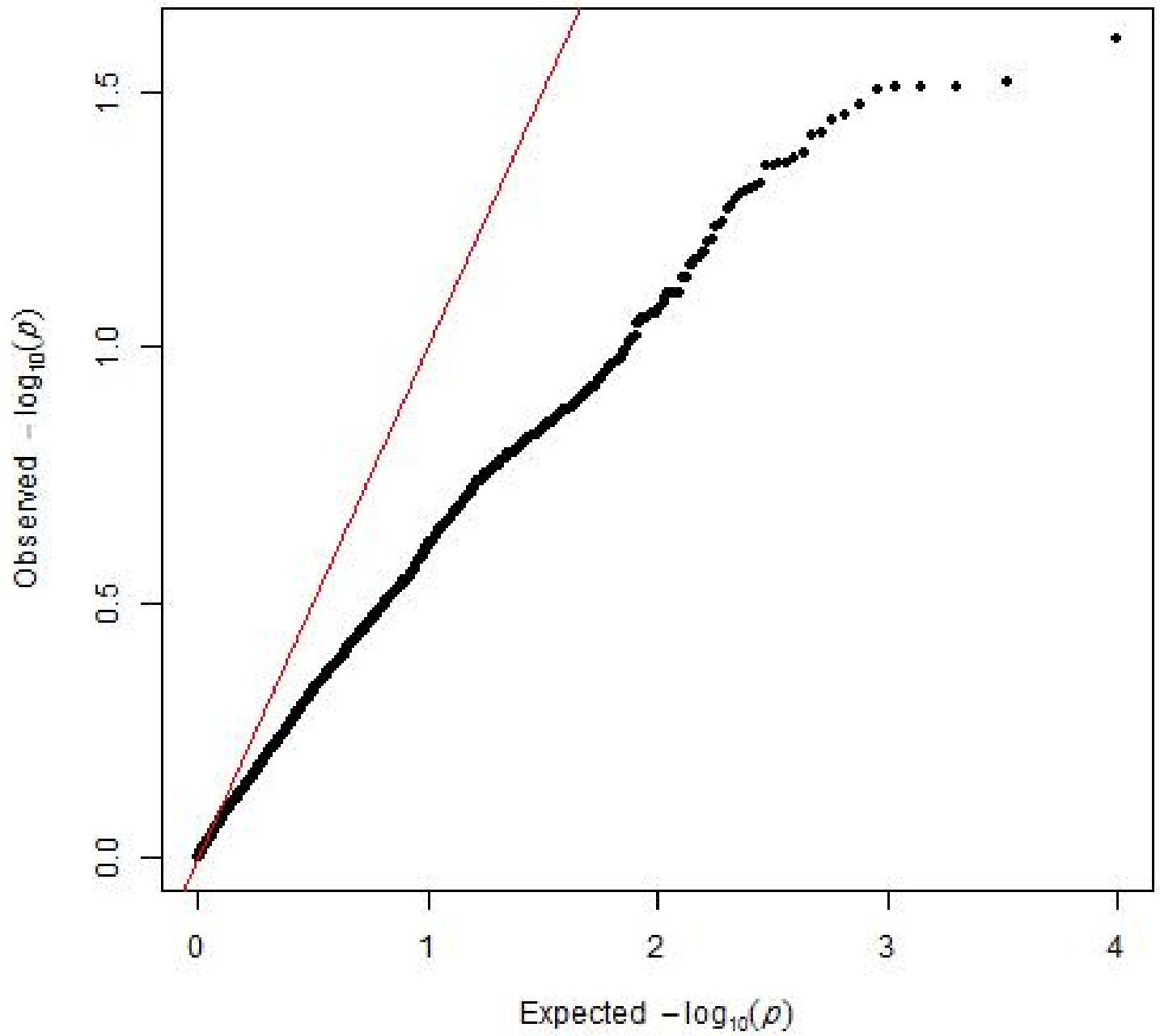

**MLM-Q**

AL

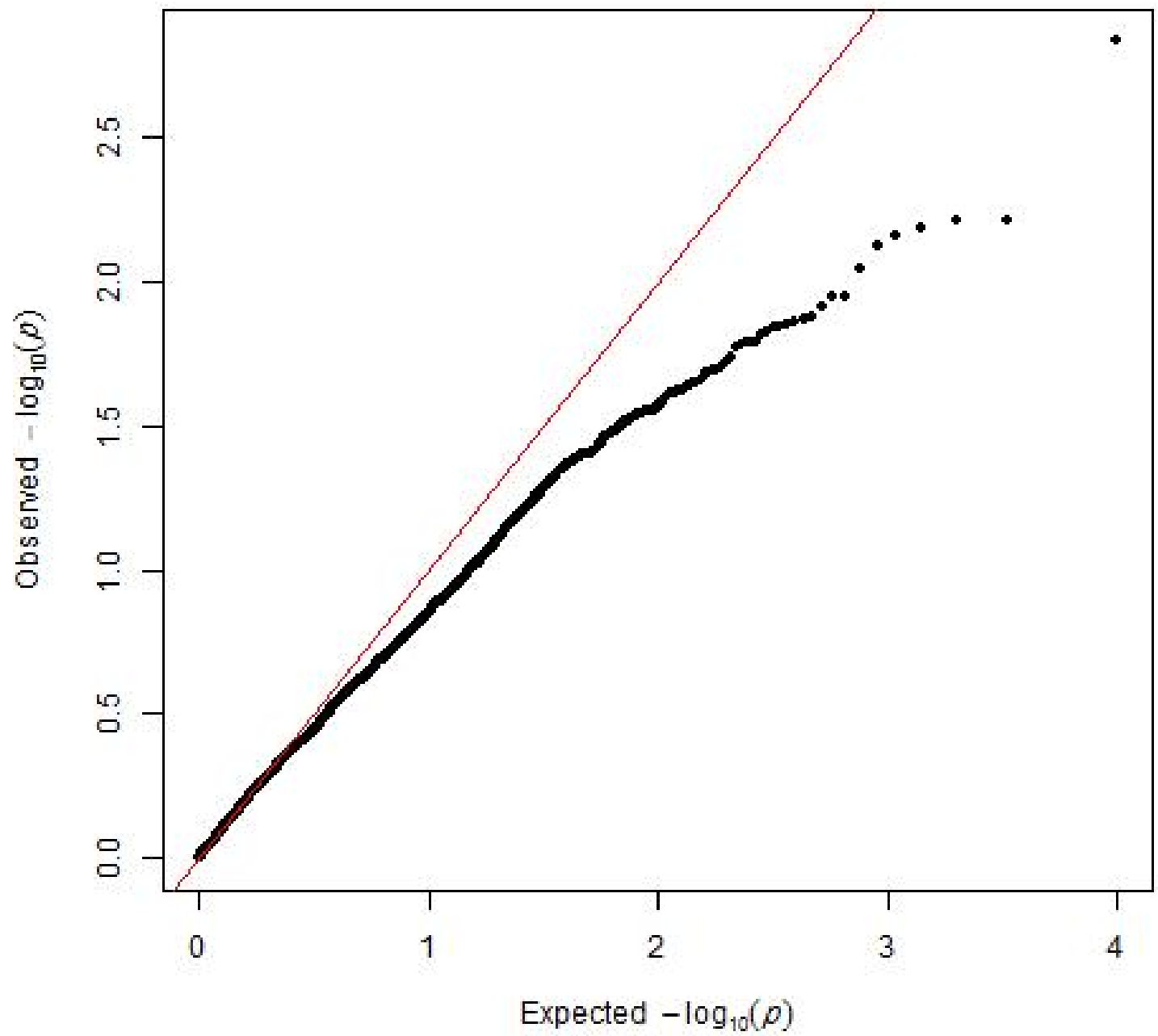

BY

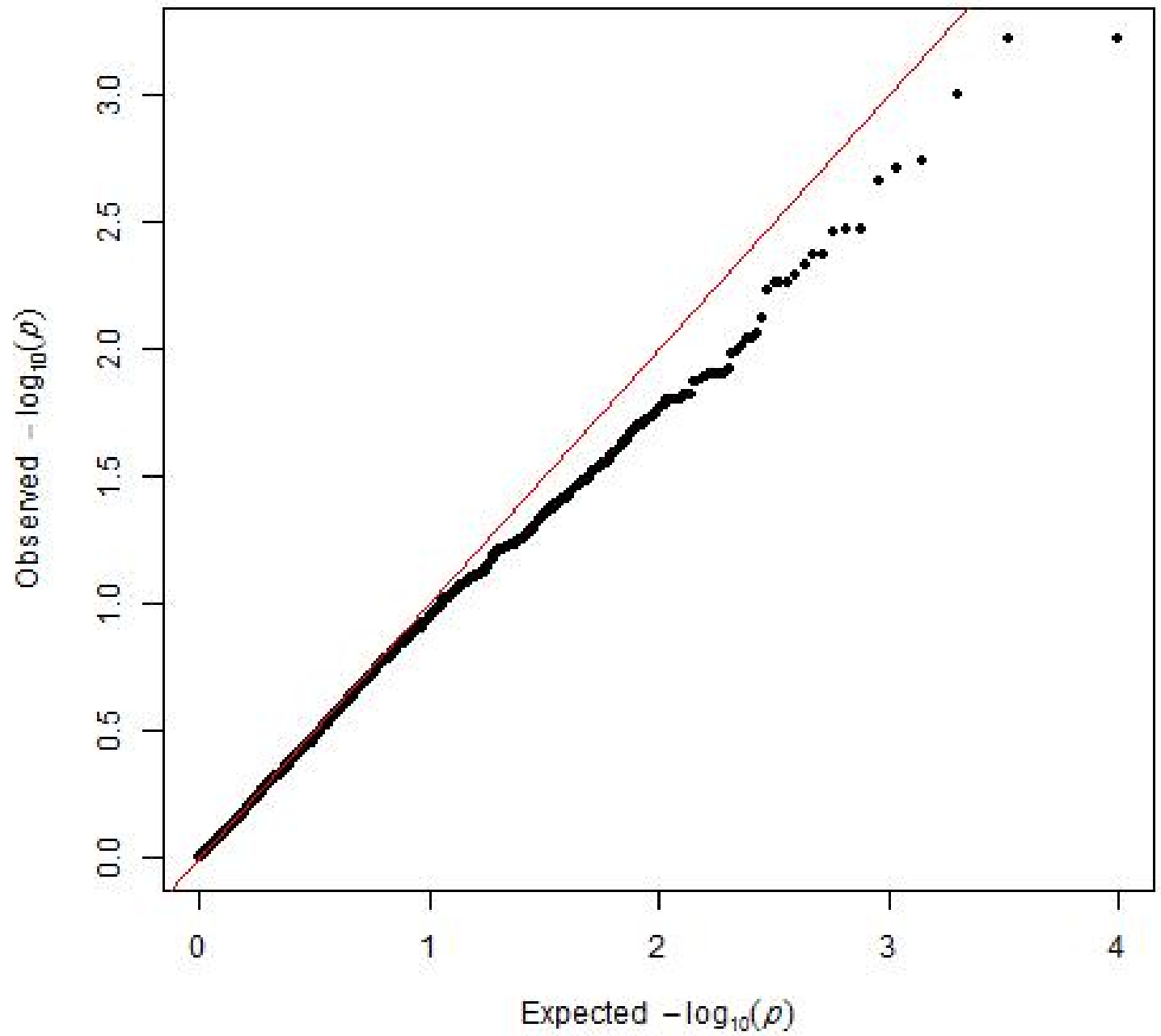

GY

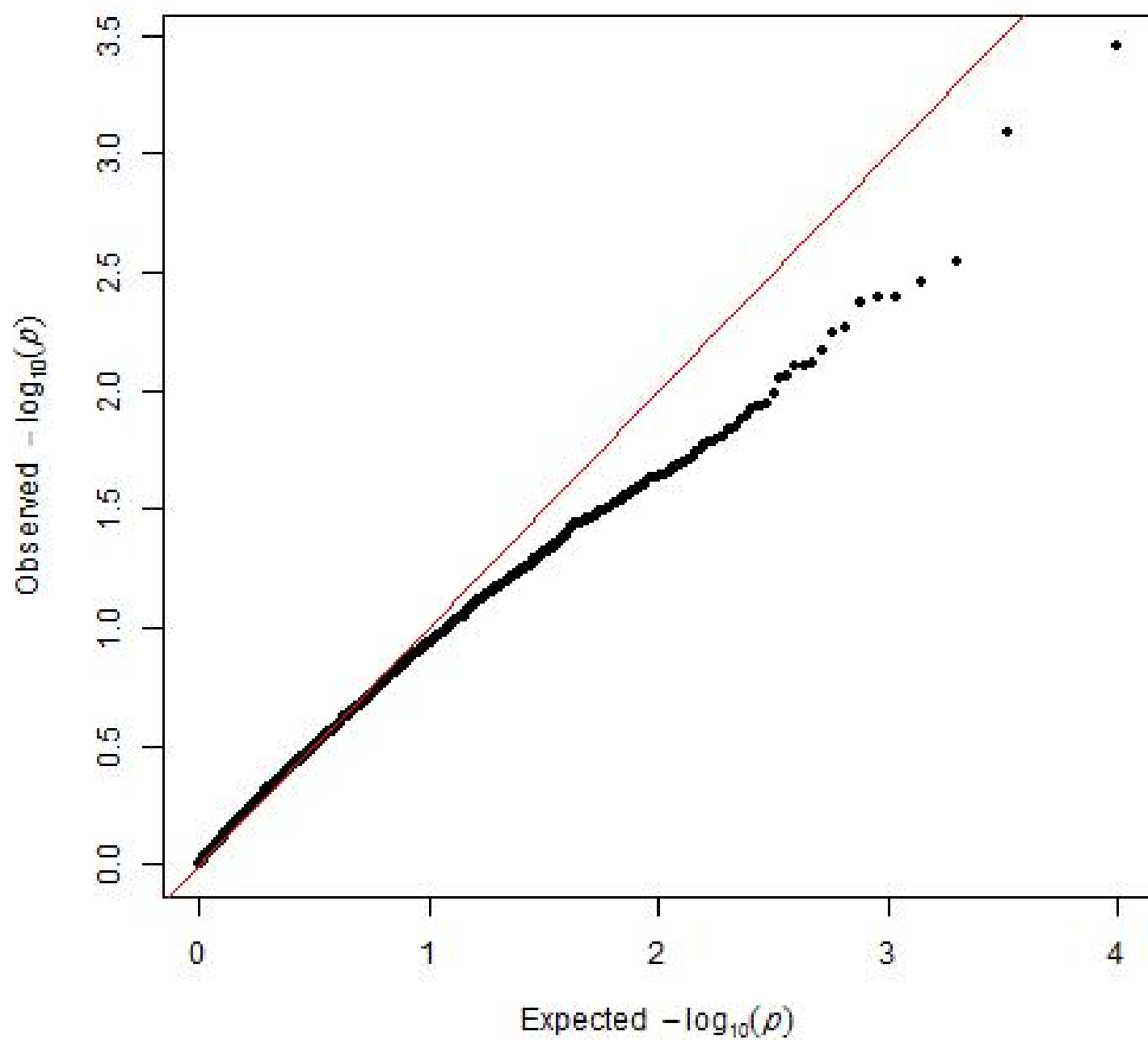

HI

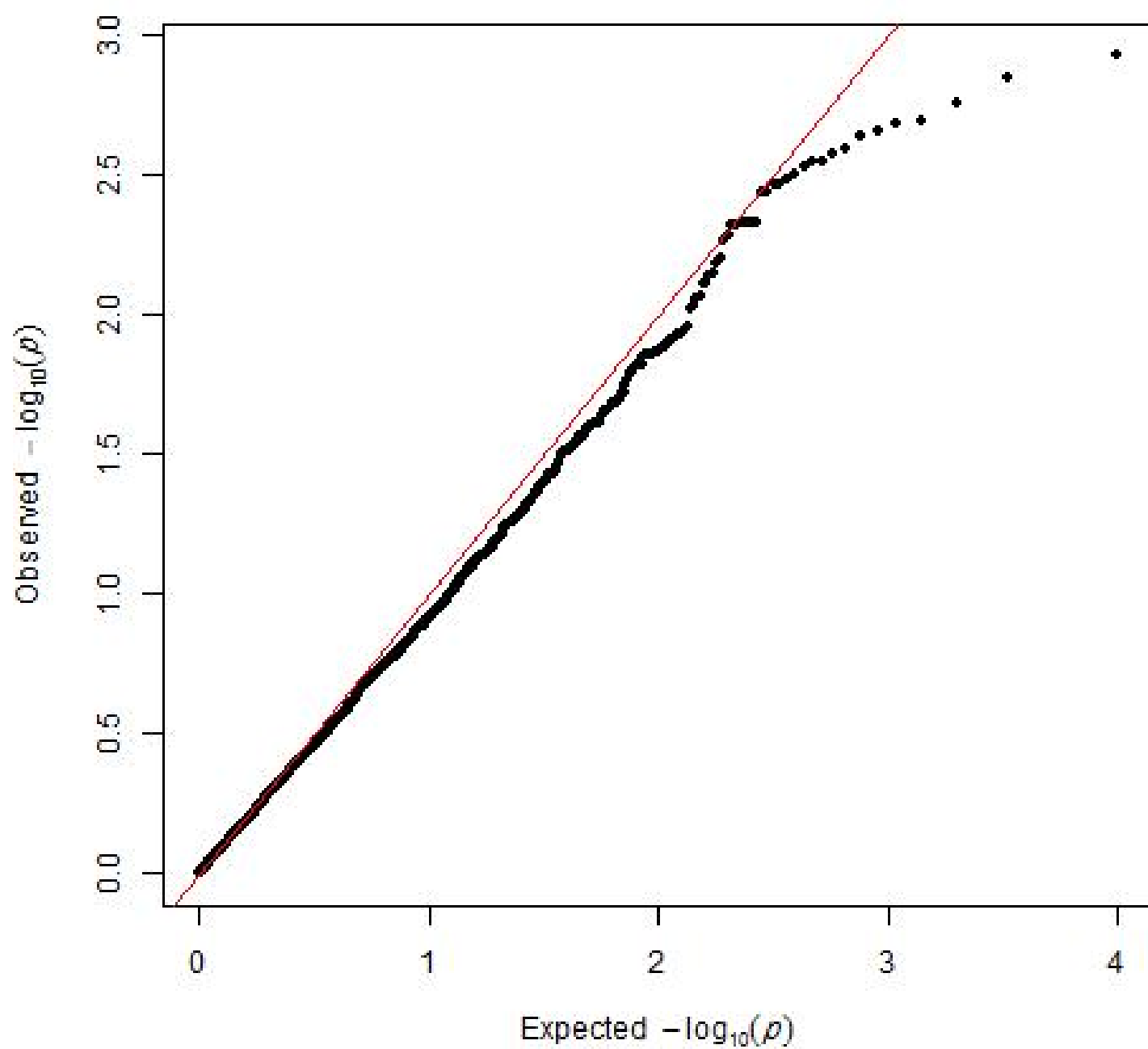

K-S

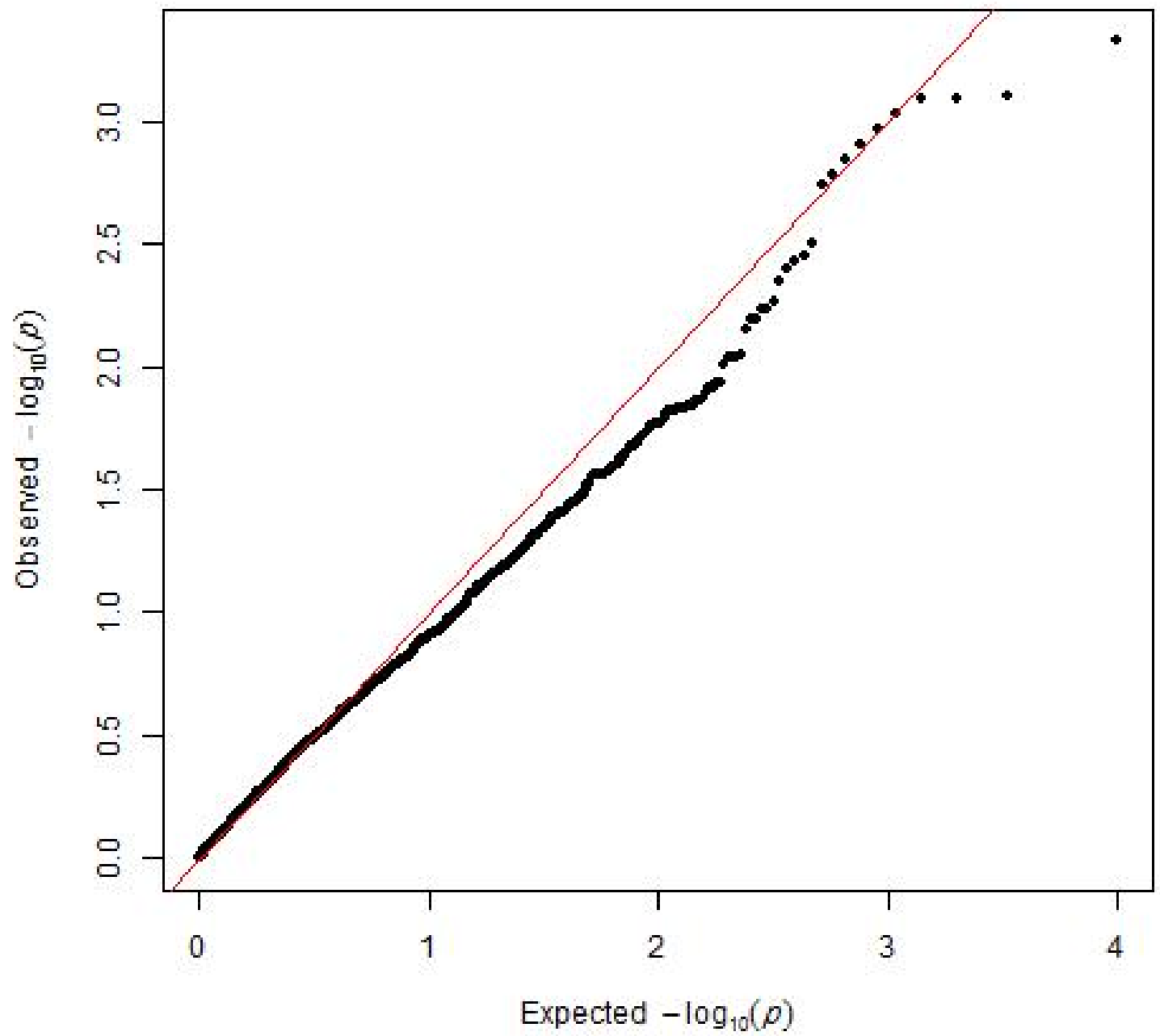

# PEX

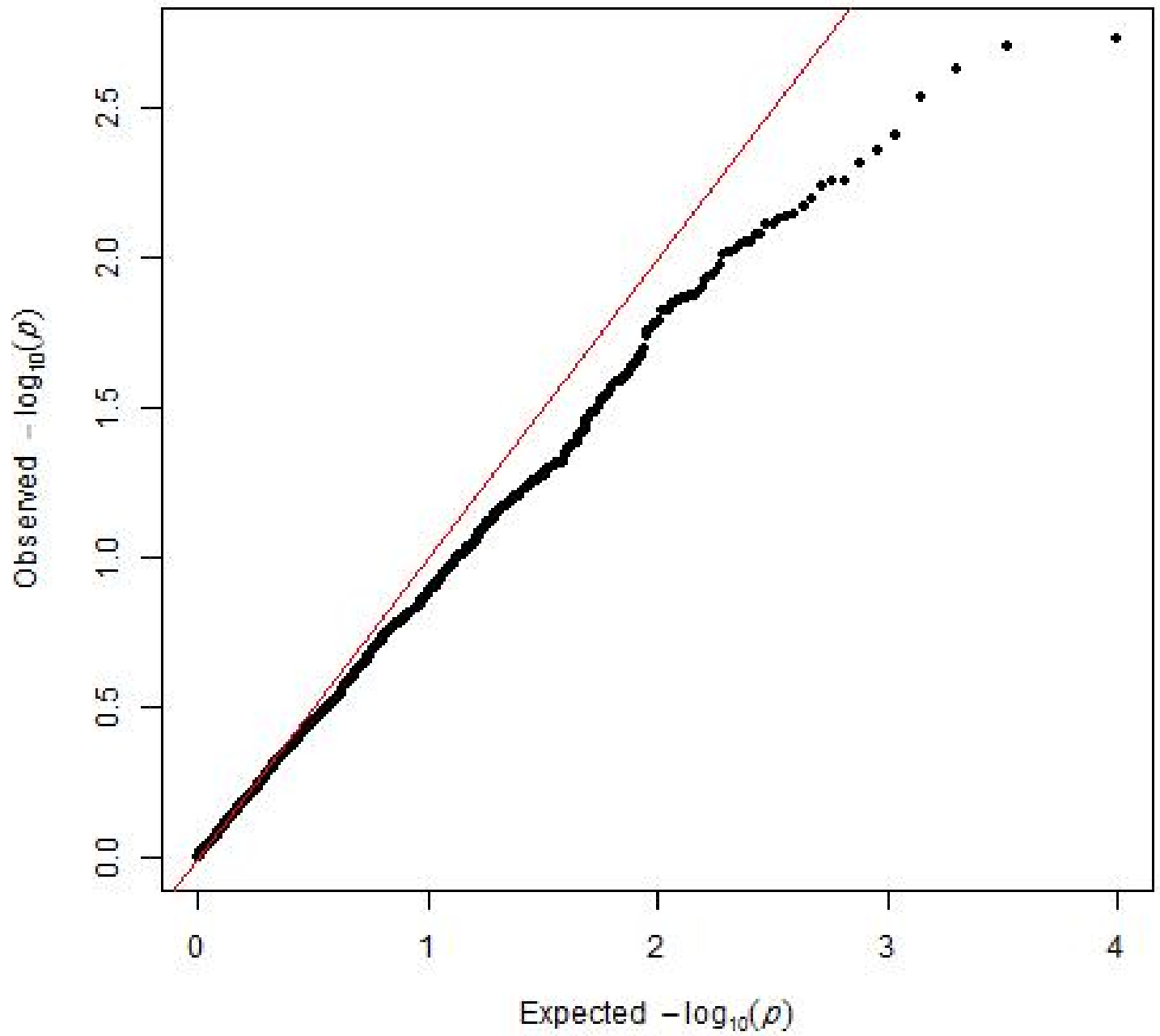

PH

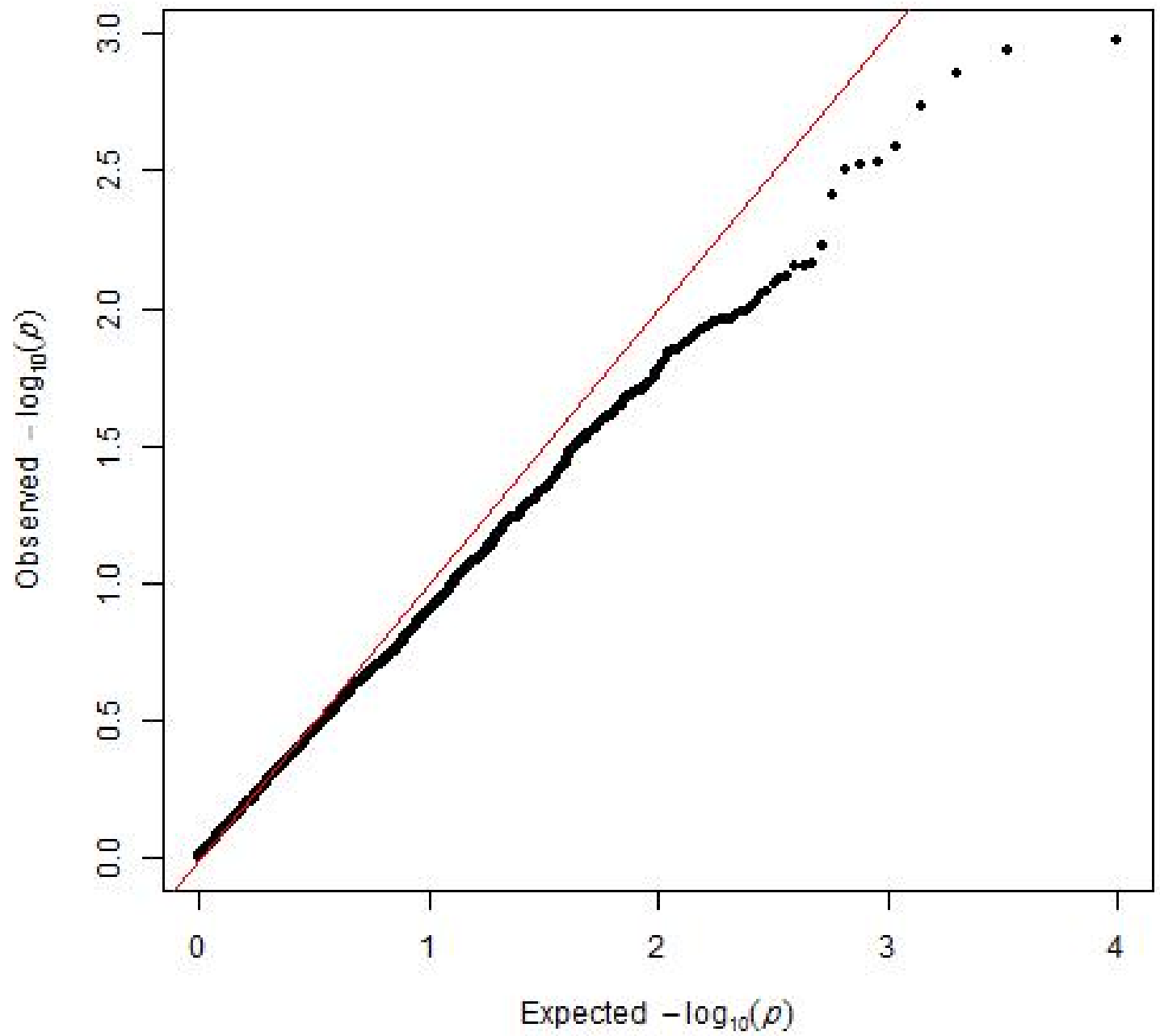

PL

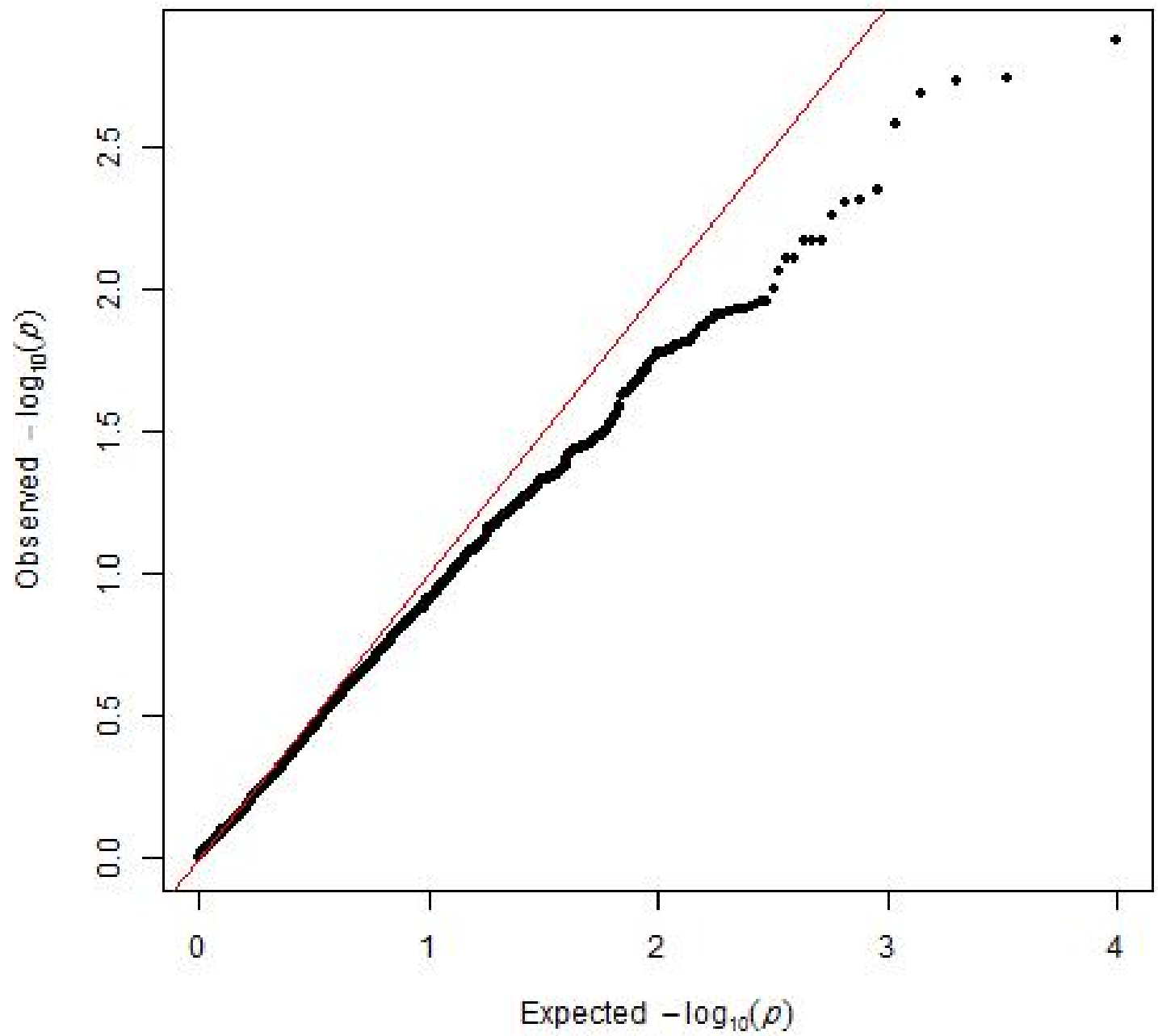

SL

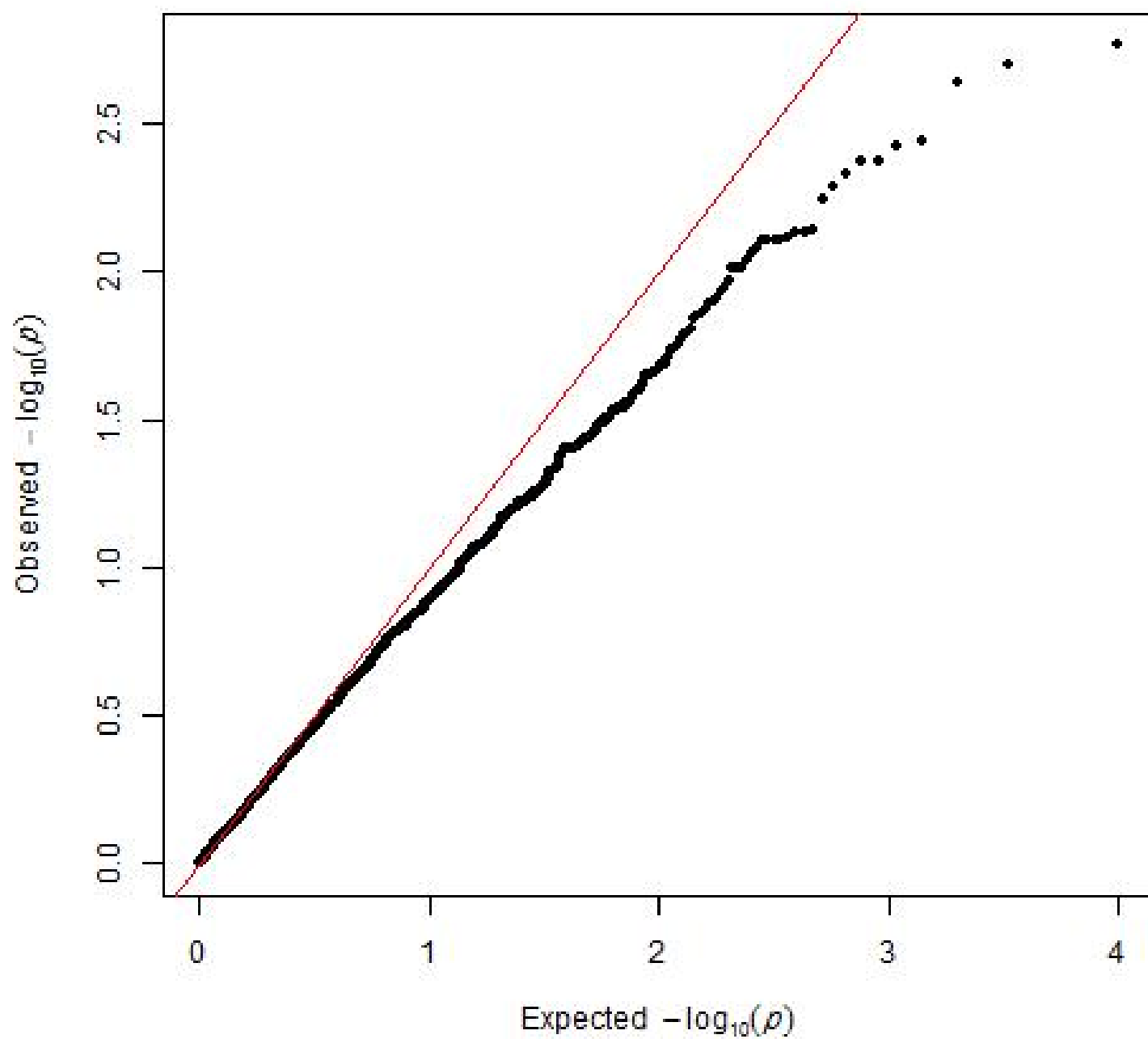

StY

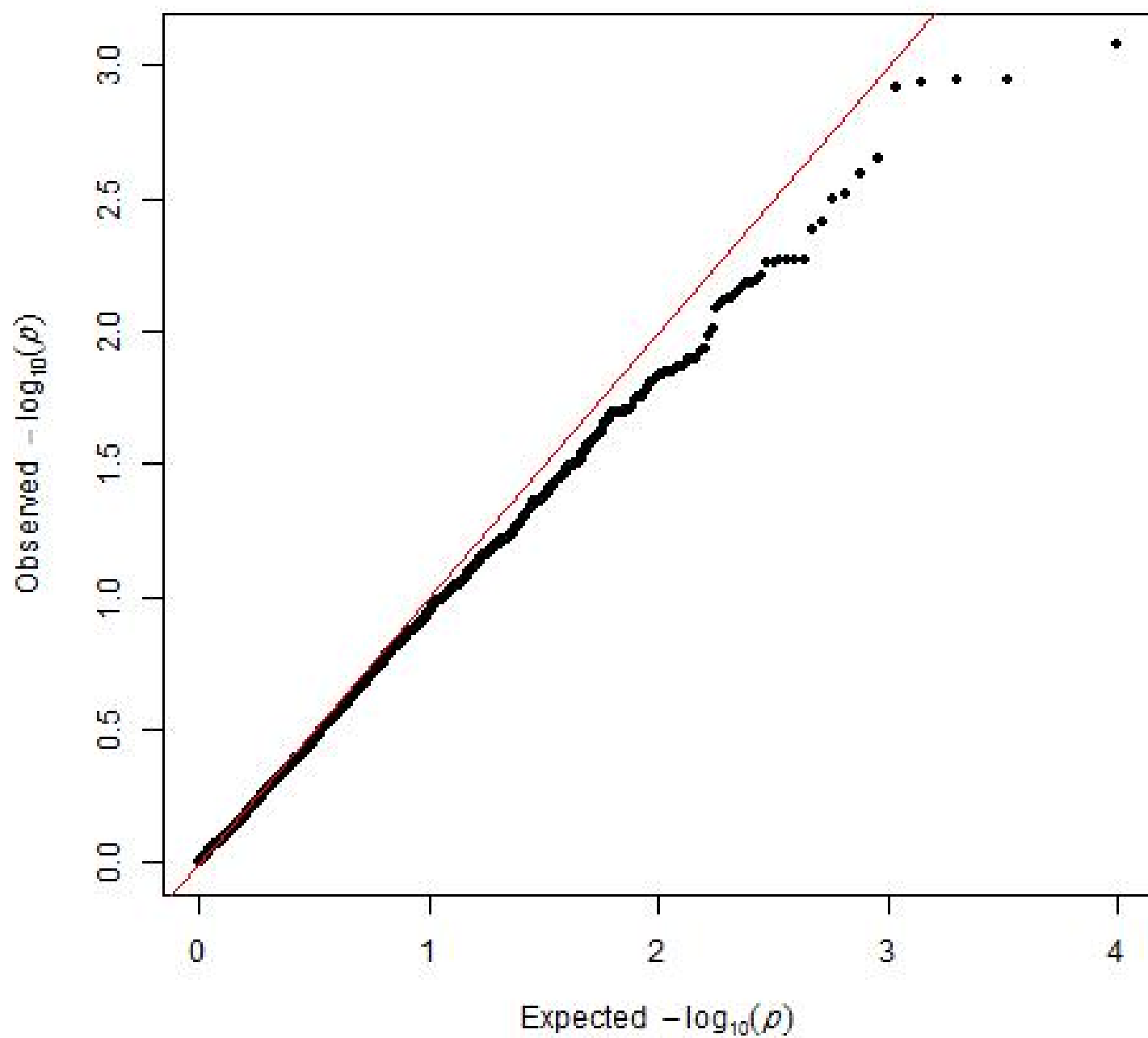

SW

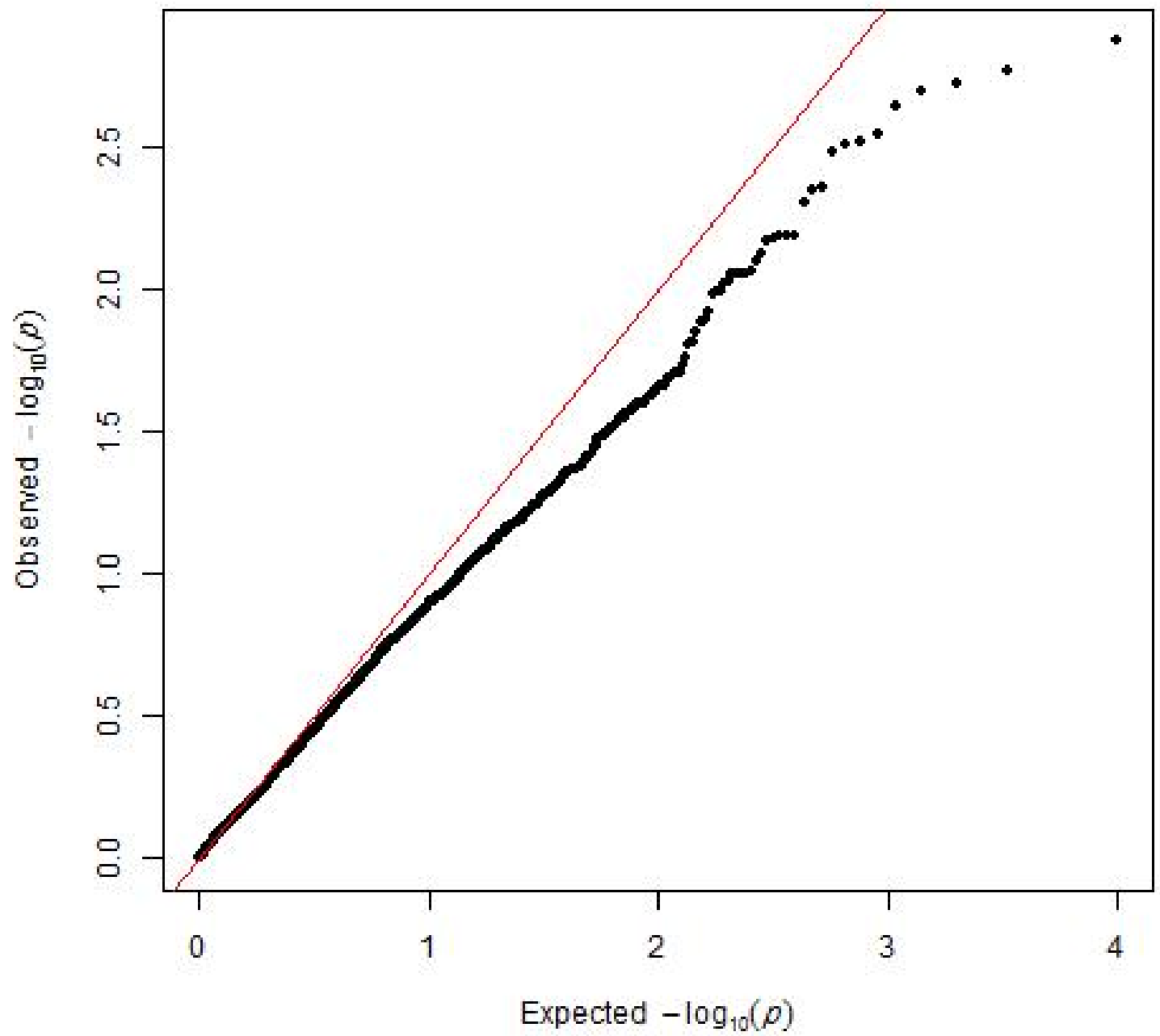

TKWT

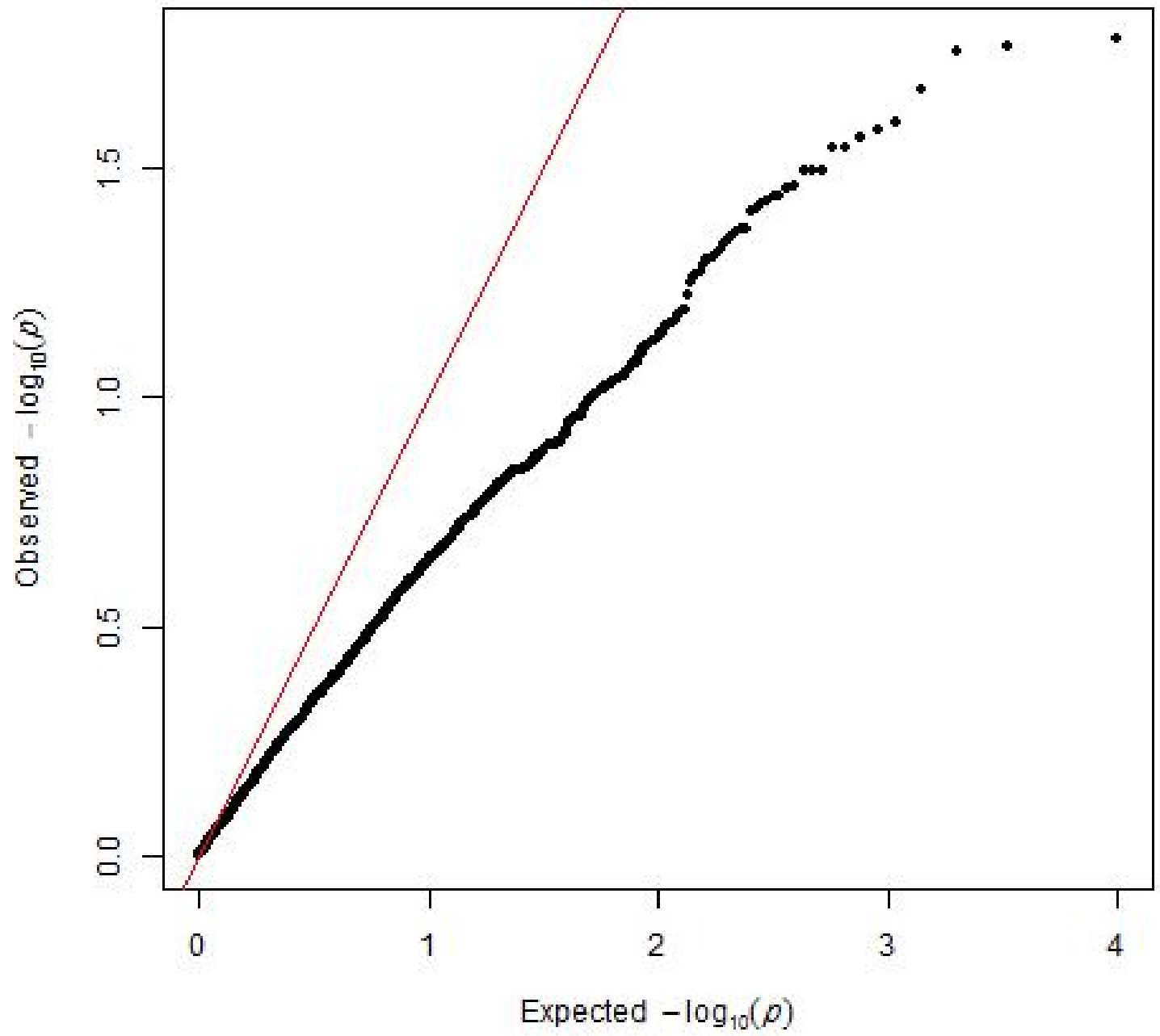

Supplement: Supplementary file 10 — QQ plots for all tested triats in this study. (PDF 360 kb) [file 12870_2017_1140_MOESM10_ESM.pdf]
